# METTL5 deficiency impairs osteogenesis through OSER1-dependent antioxidant regulation

**DOI:** 10.1172/jci.insight.194068

**Published:** 2026-05-08

**Authors:** Kexin Lei, Qi Yin, Qiwen Li, Qian Wang, Zhong Zhang, Fei Xue, Ruoshi Xu, Xinyi Zhou, Lin Peng, Shoichiro Kokabu, Shuibin Lin, Quan Yuan

**Affiliations:** 1 State Key Laboratory of Oral Diseases, National Center for Stomatology, and National Clinical Research Center for Oral Diseases, West China Hospital of Stomatology, Sichuan University, Chengdu, China.; 2 Department of Prosthodontics, Shanghai Ninth People’s Hospital, Shanghai Jiao Tong University School of Medicine, Shanghai, China.; 3 Division of Molecular Signaling and Biochemistry, Kyushu Dental University, Kitakyushu, Fukuoka, Japan.; 4 Center for Translational Medicine, Precision Medicine Institute, The First Affiliated Hospital, Sun Yat-sen University, Guangzhou, China.

**Keywords:** Bone biology, Cell biology, Bone disease, Genetic diseases, Stem Cells

## Abstract

Methyltransferase-like 5 (METTL5) is a methyltransferase responsible for rRNA N6-methyladenosine (m^6^A) modification, mutations in which are associated with skeletal abnormalities and cognitive deficits. Despite METTL5’s clinical relevance, the molecular mechanisms underlying METTL5-related genetic disorders remain poorly understood. In this study, we demonstrated that *Mettl5* KO led to reduced bone mass and smaller body size in mice and impaired the osteogenic differentiation of mesenchymal stem cells. Mechanistically, *Mettl5* deficiency decreased the translation efficiency of oxidative stress–responsive serine-rich protein 1 mRNA, downregulated the expression of key antioxidant genes, and diminished antioxidant capacity. Importantly, administration of the antioxidant N-acetylcysteine (NAC) partially rescued skeletal defects in *Mettl5*-KO mice. These findings reveal a critical role for METTL5 in antioxidant defense and suggest that NAC supplementation may represent a promising therapeutic strategy for METTL5-related disorders.

## Abstract 2

METTL5 maintains bone health through antioxidant regulation and its loss leads to bone loss, while antioxidant therapy restores bone mass, offering hope for METTL5-related disorders.

## Graphical abstract

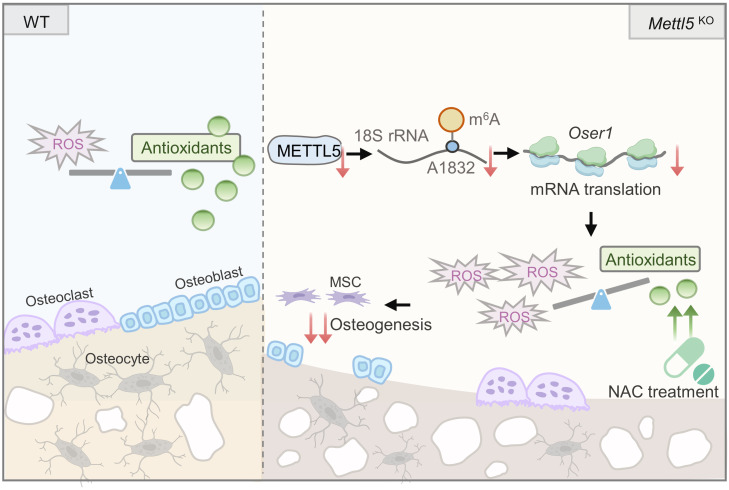

## Introduction

RNA N6-methyladenosine (m^6^A) modification is a key posttranscriptional modification found in various RNA species, including mRNA and rRNA. In rRNA, m^6^A modification occurs at position 1832 of 18S rRNA and position 4220 of 28S rRNA (1, 2). Methyltransferase-like 5 (METTL5) is the methyltransferase responsible for catalyzing the m^6^A modification at the A1832 site of eukaryotic 18S rRNA. It plays important roles in regulating translation efficiency, fatty acid metabolism, cell differentiation, and cell proliferation (3–6). Dysregulation of METTL5 has been implicated in multiple diseases, including cancer, where it is often upregulated and associated with poor prognosis (4, 7). Furthermore, exome sequencing has identified *METTL5* as a causal gene in autosomal recessive intellectual disability, with reported mutations associated with short stature, craniofacial malformations, microcephaly, and behavioral abnormalities (8–11). Animal studies further support a developmental role for METTL5: knockdown of *Mettl5* in zebrafish embryos reduces head size, whereas *Mettl5* deletion in mice delays cranial suture closure (10, 12). Despite these findings, the molecular mechanisms underlying METTL5-associated skeletal defects remain poorly understood.

Bone marrow mesenchymal stem cells (MSCs) are multipotent stem cells residing in the BM that contribute to bone formation and remodeling (13). MSCs differentiate into osteoblasts and directly contribute to bone formation, thereby playing a central role in skeletal homeostasis (14). Among the diverse mechanisms governing these processes, ROS have emerged as critical modulators. ROS are primarily generated by mitochondrial complexes I and III, as well as by the NADPH oxidase isoform NOX4. Physiological levels of ROS contribute to normal differentiation and signaling, whereas excessive oxidative stress can damage DNA, proteins, and lipids under pathological conditions (15, 16). Specifically, elevated ROS levels directly suppress osteogenic differentiation in osteoblast-lineage cells and MSCs, resulting in reduced osteogenic activity and impaired matrix mineralization (17, 18). Conversely, antioxidant interventions partially restore osteogenic differentiation under oxidative stress conditions, supporting a direct regulatory role of redox balance in osteogenesis (19). FOXO transcription factors act as key regulators of oxidative stress, and their deletion in mice leads to elevated ROS levels within bone tissue and subsequent bone loss (20, 21). Mechanistically, excessive ROS can inhibit osteoblast proliferation and differentiation by suppressing the Wnt/β-catenin pathway and activating the c-JNK pathway (22, 23) and may also influence bone remodeling via activation of NF-κB (24, 25). Collectively, these findings highlight oxidative stress as a major determinant of bone homeostasis, and targeting ROS regulation has emerged as a promising therapeutic strategy.

To counteract oxidative stress, cells rely on antioxidants, which are crucial for maintaining redox homeostasis and supporting osteogenic differentiation. Accumulating evidence indicates that antioxidants promote osteogenic differentiation and improve bone homeostasis under oxidative stress conditions (26, 27). They enhance bone formation and attenuate inflammation by modulating key signaling pathways, including NF-κB, MAPK, PI3K/AKT, and Wnt/β-catenin (24, 28–32). Under pathological conditions such as osteoporosis and diabetic bone defects, antioxidants improve the bone microenvironment by reducing ROS levels, thereby facilitating bone repair and increasing bone mineral density (33, 34). Consistent with experimental findings, a population-based cross-sectional study demonstrated that total dietary antioxidant capacity is inversely associated with osteoporosis risk in postmenopausal women and positively correlated with bone mass in both pre- and postmenopausal women (35). Collectively, these studies underscore the essential role of antioxidant defense in bone biology and diseases.

Interestingly, recent studies have implicated METTL5 in the regulation of stress-responsive genes, contributing to the cellular adaptation to external stimuli (36, 37). Most research to date has focused on its involvement in the integrated stress response and in responses to general stimuli such as UV radiation and heat shock (36, 37). However, whether METTL5 directly influences oxidative stress and antioxidant regulation in osteogenic lineage cells remains unknown. Motivated by the skeletal abnormalities observed in individuals carrying *METTL5* mutations, we aimed to investigate whether METTL5 plays a critical role in bone development and homeostasis. We hypothesized that METTL5 deficiency impairs osteogenesis, potentially via disrupted antioxidant defense and redox imbalance, thereby contributing to skeletal abnormalities.

## Results

### Deletion of Mettl5 leads to reduced bone mass in mice and impaired osteogenic differentiation of MSCs.

**Figure 1:**
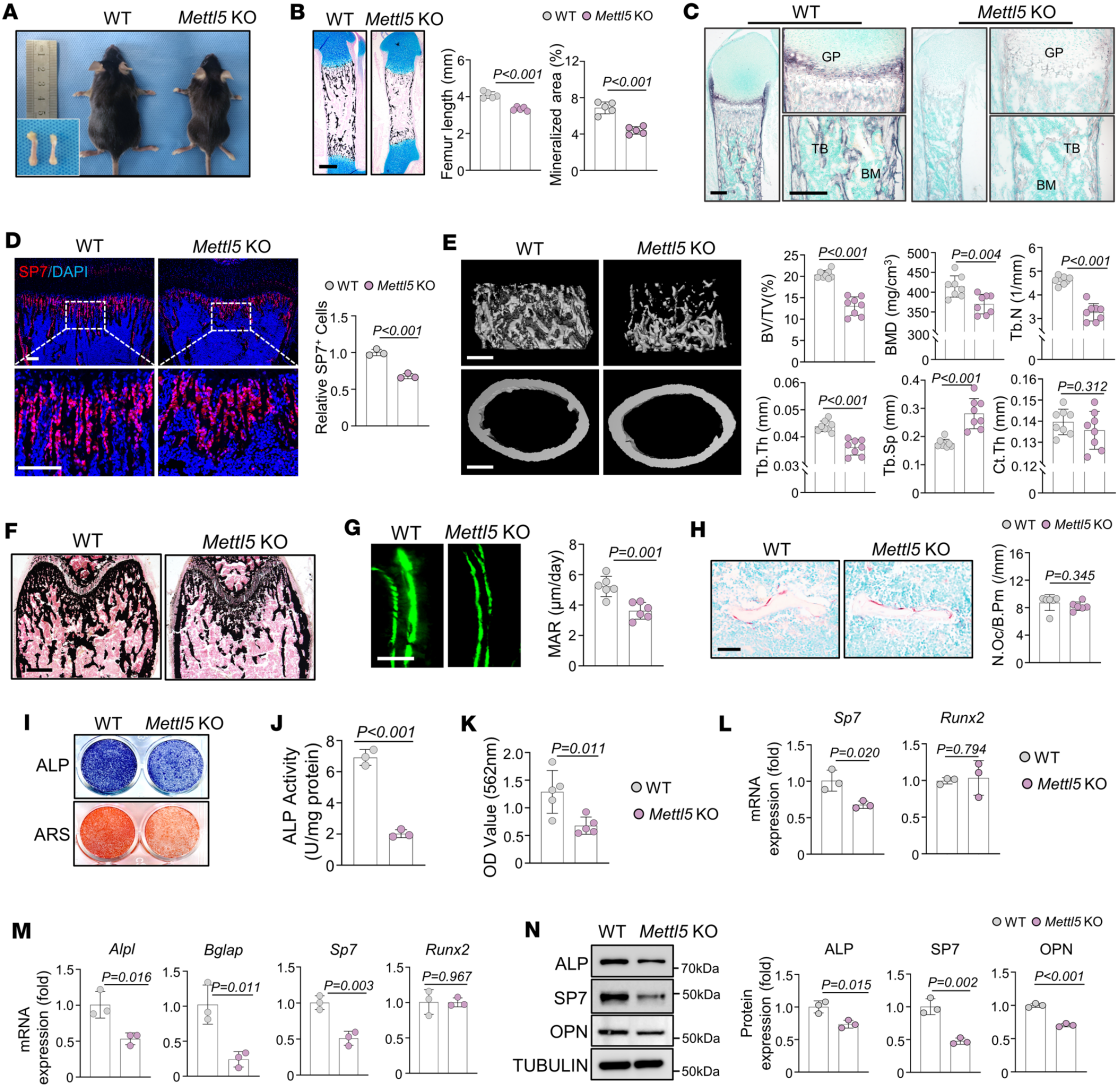
*Mettl5* deficiency leads to reduced bone mass and impaired osteogenic differentiation. (**A**) Gross appearance and femoral morphology of 6-week-old male WT and *Mettl5*-KO mice. *n* = 8. (**B**) Von Kossa staining of femurs at P1, with quantification of femur length and mineralized area. Scale bar: 400 μm. *n* = 5. (**C**) ALP activity staining of P1 femurs from WT and *Mettl5-*KO mice. GP, growth plate; TB, trabecular bone. Scale bar: 200 μm. *n* = 3. (**D**) Representative immunofluorescence images and quantification of SP7 staining in femurs from P14 WT and *Mettl5-*KO mice, with magnified views of the boxed regions. Scale bar: 100 μm. *n* = 3. (**E**) MicroCT analysis of trabecular bone and cortical bone in femurs from 6-week-old male WT and *Mettl5-*KO mice. Scale bar: 400 μm. *n* = 8. (**F**) Representative Von Kossa staining of femurs from 6-week-old male WT and *Mettl5-*KO mice. Scale bar: 400 μm. *n* = 5. (**G**) Representative Calcein labeling images and quantification showing the mineral apposition rate (MAR). Scale bar: 50 μm. *n* = 6. (**H**) Representative TRAP staining of femurs from 6-week-old male WT and *Mettl5-*KO mice, with quantification of osteoclast number per bone perimeter (N.Oc/B.Pm). Scale bar: 50 μm. *n* = 6. (**I**) Representative images of ALP and ARS staining in MSCs derived from WT and *Mettl5-*KO mice after osteogenic induction. *n* = 5. (**J** and **K**) Quantification of ALP activity (*n* = 3) and ARS staining (*n* = 5) in MSCs derived from WT and *Mettl5-*KO mice. (**L**) qRT-PCR analysis of *Sp7* and *Runx2* in MSCs from WT and *Mettl5-*KO mice after 3 days of osteogenic induction. *n* = 3. (**M**) qRT-PCR analysis of osteogenic markers in MSCs from WT and *Mettl5-*KO mice after 5 days of osteogenic induction. *n* = 3. (**N**) Representative Western blot images and quantifications showing the protein levels of osteogenic markers in MSCs from WT and *Mettl5-*KO mice after osteogenic induction. *n* = 3. Data are expressed as mean ± SD; *P* values were determined by 2-tailed Student’s *t* test.

As previously reported, whole-body *Mettl5*-KO mice showed a reduced birth rate but remained viable (12). Consistent with these findings, *Mettl5-*KO mice also exhibited shorter body length and smaller body size compared with WT controls (Figure 1A). IHC staining of neonatal WT femurs showed that METTL5 was expressed in resting and hypertrophic chondrocytes as well as in the BM (Supplemental Figure 1A; supplemental material available online with this article; https://doi.org/10.1172/jci.insight.194068DS1). Von Kossa staining of neonatal *Mettl5-*KO mice revealed decreased femur length and reduced mineralized area relative to WT controls (Figure 1B). In addition, alkaline phosphatase (ALP) staining was markedly reduced in the growth plate, periosteum, and trabecular bone of *Mettl5-*KO mice (Figure 1C), and the number of SP7-positive osteoblasts beneath the growth plate was reduced, indicating impaired osteogenic capacity compared with WT controls (Figure 1D). MicroCT analysis of femurs from 6-week-old male mice revealed compromised trabecular bone mass in *Mettl5-*KO mice, characterized by decreases in trabecular bone volume per tissue volume, bone mineral density, trabecular number, and trabecular thickness, together with increased trabecular separation, whereas cortical bone thickness was not significantly altered (Figure 1E). Consistently, Von Kossa staining of femurs from 6-week-old male mice showed reduced mineralized tissue in *Mettl5-*KO mice (Figure 1F). Dynamic bone formation assessed by Calcein double-labeling revealed a reduced mineral apposition rate in trabecular bone of *Mettl5-*KO mice, indicating slower bone formation (Figure 1G). Tartrate-resistant acid phosphatase (TRAP) staining showed no significant difference in osteoclast numbers between *Mettl5-*KO and WT mice (Figure 1H). *Mettl5* mRNA exhibited a dynamic expression pattern during osteogenic differentiation of MC3T3-E1 cells, with a transient increase at day 3 followed by stabilization at days 5 and 7 (Supplemental Figure 1B). To investigate the effect of *Mettl5* deletion on osteogenic differentiation of MSCs, primary BM MSCs were isolated from *Mettl5-*KO mice and littermate controls. Upon osteogenic induction, *Mettl5*-deficient MSCs exhibited reduced ALP activity and fewer mineralized nodules compared with WT controls (Figure 1, I–K). Quantitative reverse transcription PCR (qRT-PCR) analysis further revealed that *Sp7* expression was already reduced in *Mettl5-*KO MSCs at an early stage of osteogenic induction (day 3), whereas *Runx2* expression remained unchanged (Figure 1L). At a later stage (day 5), decreased mRNA expression of osteogenic markers, including *Alpl*, *Bglap*, and *Sp7*, was observed, accompanied by reduced protein levels of ALP, SP7, and osteopontin in *Mettl5-*KO MSCs (Figure 1, M and N).

### Deletion of Mettl5 inhibits bone formation and osteogenic differentiation in limb mesenchyme–specific KO mice.

**Figure 2:**
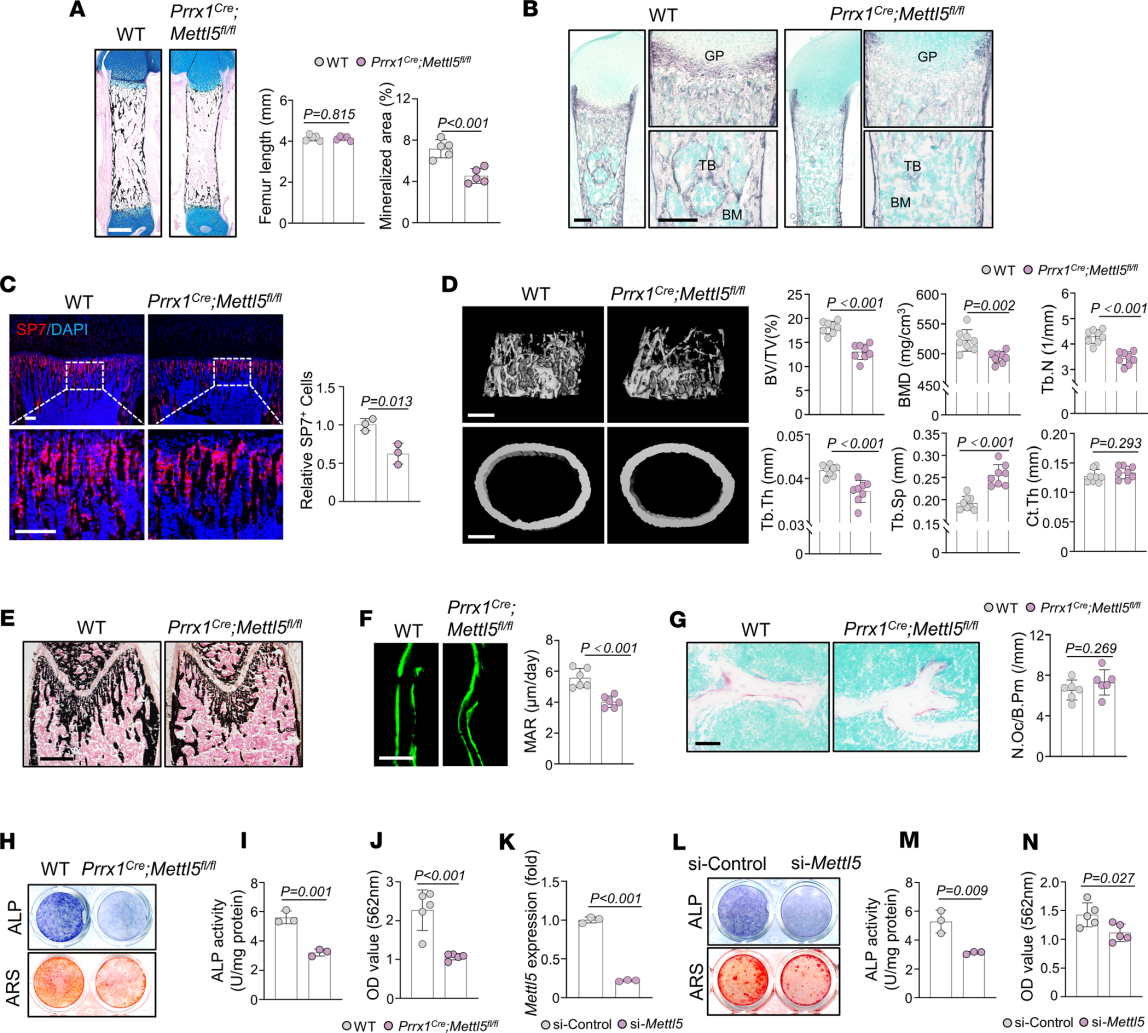
Deletion of *Mettl5* impairs osteogenesis in limb mesenchyme–specific KO mice. (**A**) Von Kossa staining of femurs from P1 WT and *Prrx1^Cre^ Mettl5^fl/fl^* mice, with quantification of femur length and mineralized area. Scale bar: 400 μm. *n* = 5. (**B**) ALP activity staining of femurs from P1 WT and *Prrx1^Cre^ Mettl5^fl/fl^* mice, with magnified views of growth plate and trabecular bone regions. GP, growth plate; TB, trabecular bone. Scale bar: 200 μm. *n* = 3. (**C**) Representative immunofluorescence images and quantification of SP7 staining in femurs from P14 WT and *Prrx1^Cre^ Mettl5^fl/fl^* mice, with magnified views (boxed regions). Scale bar: 100 μm. *n* = 3. (**D**) MicroCT reconstructions of trabecular bone (top) and cortical bone (bottom) in femurs from 6-week-old male WT and *Prrx1^Cre^ Mettl5^fl/fl^* mice with quantitative analyses. Scale bar: 400 μm. *n* = 8. (**E**) Representative Von Kossa staining of femurs from 6-week-old male WT and *Prrx1^Cre^ Mettl5^fl/fl^* mice. Scale bar: 400 μm. *n* = 5. (**F**) Representative Calcein labeling images and quantitative analysis of mineral apposition rate (MAR) in femurs from 6-week-old male WT and *Prrx1^Cre^ Mettl5^fl/fl^* mice. Scale bar: 50 μm. *n* = 6. (**G**) Representative TRAP staining of femurs from 6-week-old male WT and *Prrx1^Cre^ Mettl5^fl/fl^* mice with quantification. Scale bar: 50 μm. *n* = 6. (**H**) Representative images of ALP and ARS staining in MSCs derived from WT and *Prrx1^Cre^ Mettl5^fl/fl^* mice after osteogenic induction. *n* = 5. (**I** and **J**) Quantification of ALP activity (*n* = 3) and ARS staining (*n* = 5) in MSCs derived from WT and *Prrx1^Cre^ Mettl5^fl/fl^* mice. (**K**) qRT-PCR validation of *Mettl5* knockdown efficiency using siRNA in MC3T3-E1 cells. *n* = 3. (**L**) Representative images of ALP and ARS staining in MC3T3-E1 cells transfected with si-Control or si-*Mettl5* after osteogenic induction. *n* = 5. (**M** and **N**) Quantification of ALP activity (*n* = 3) and ARS staining (*n* = 5) in MC3T3-E1 cells transfected with si-Control or si-*Mettl5*. Data shown as mean ± SD; *P* values determined by 2-tailed Student’s *t* test.

To further validate the role of METTL5 in limb mesenchymal and osteogenic progenitors, we generated limb mesenchyme–specific *Mettl5-*KO mice by crossing *Mettl5^fl/fl^* mice with *Prrx1^Cre^* mice. Von Kossa staining of neonatal *Prrx1^Cre^ Mettl5^fl/fl^* mice revealed decreased mineralized area, although femur length was comparable to that of controls (Figure 2A). Additionally, *Prrx1^Cre^ Mettl5^fl/fl^* mice exhibited reduced ALP staining and fewer SP7-positive cells beneath the growth plate (Figure 2, B and C). MicroCT analysis and Von Kossa staining of femurs from 6-week-old male mice confirmed reduced trabecular bone mass in *Prrx1^Cre^ Mettl5^fl/fl^* mice, while cortical bone thickness remained comparable to controls (Figure 2, D and E). Dynamic bone formation assessed by Calcein double-labeling revealed a decreased mineral apposition rate in *Prrx1^Cre^ Mettl5^fl/fl^* mice, indicating impaired bone formation compared with controls (Figure 2F). TRAP staining revealed no significant difference in osteoclast numbers between the 2 groups (Figure 2G). Primary MSCs were isolated from *Prrx1^Cre^ Mettl5^fl/fl^* mice and control littermates and subjected to osteogenic induction in vitro. *Prrx1^Cre^ Mettl5^fl/fl^* MSCs exhibited decreased ALP activity and diminished mineralized nodule formation (Figure 2, H–J). In MC3T3-E1 cells, siRNA-mediated knockdown of *Mettl5* was confirmed by qRT-PCR (Figure 2K) and similarly impaired osteogenic differentiation, as evidenced by reduced ALP activity and matrix mineralization (Figure 2, L–N).

### Deletion of Mettl5 does not affect osteoclast differentiation.

To evaluate whether *Mettl5* deletion affects osteoclast differentiation, primary BM-derived macrophages (BMDMs) were isolated and induced toward the osteoclast lineage. After induction, TRAP staining revealed no significant differences in osteoclast numbers or size between the *Mettl5-*KO and control groups (Supplemental Figure 2, A and B). Likewise, qRT-PCR analysis showed no significant changes in the mRNA expression of osteoclast marker genes (Supplemental Figure 2C).

To further assess the contribution of osteoclasts to the bone phenotype of *Mettl5*-deficient mice, we generated *LysM^Cre^ Mettl5^fl/fl^* mice, in which *Mettl5* was conditionally deleted in BM macrophages. MicroCT analysis revealed no significant differences in bone parameters, including bone volume per tissue volume, bone mineral density, or trabecular thickness, between *LysM^Cre^ Mettl5^fl/fl^* and controls (Supplemental Figure 3A). Von Kossa staining also showed comparable bone mass between the groups (Supplemental Figure 3B), and histological analysis confirmed that osteoclast numbers were similar between *LysM^Cre^ Mettl5^fl/fl^* mice and control mice (Supplemental Figure 3C).

### Mettl5 KO results in reduced translation of Oser1.

**Figure 3:**
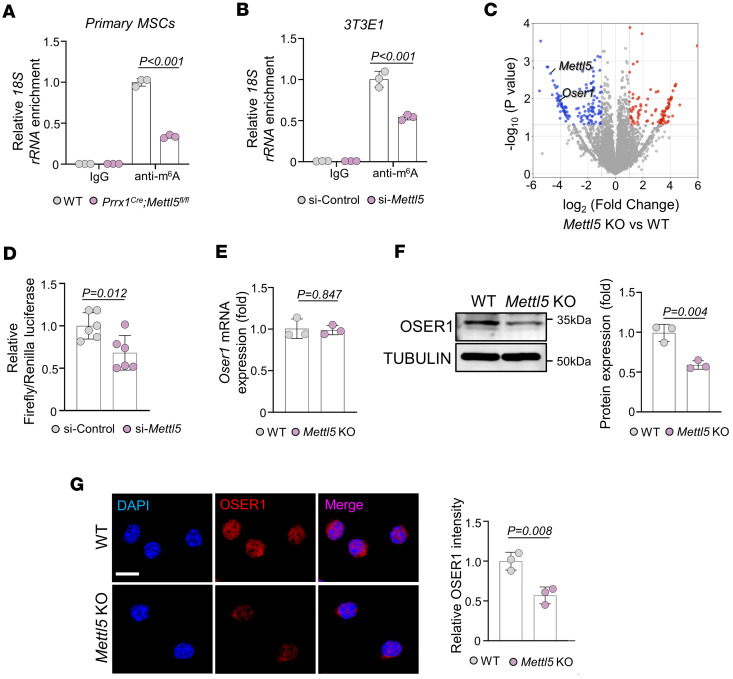
METTL5 regulates the translation of OSER1. (**A**) Methylated RNA IP–qPCR analysis of 18S rRNA m^6^A enrichment in MSCs derived from WT and *Prrx1^Cre^ Mettl5^fl/fl^* mice. *n* = 3. (**B**) Methylated RNA IP–qPCR analysis of 18S rRNA m^6^A enrichment in MC3T3-E1 cells transfected with si-Control and si-*Mettl5*. *n* = 3. (**C**) Volcano plot of differentially translated genes identified by ribosome profiling in MSCs. *Oser1* and *Mettl5* are highlighted among genes with significant altered translation efficiency. Threshold: |log_2_(FC)| > 1, *P* < 0.05. (**D**) Relative Firefly/Renilla luciferase activity in MC3T3-E1 cells transfected with an *Oser1* dual-luciferase reporter plasmid in the si-Control and si-*Mettl5* groups. *n* = 6. (**E**) qRT-PCR analysis of *Oser1* mRNA levels in MSCs from WT and *Mettl5-*KO mice. *n* = 3. (**F**) Representative Western blot images and quantification of OSER1 protein levels in WT and *Mettl5-*KO MSCs. *n* = 3. (**G**) Representative immunofluorescence images showing OSER1 expression in WT and *Mettl5-*KO MSCs, with corresponding quantification. Scale bar: 10 μm. *n* = 3. Data are expressed as mean ± SD; *P* values were determined by 2-tailed Student’s *t* test.

To validate that METTL5 mediates m^6^A modification of 18S rRNA, we performed methylated RNA IP–qPCR analysis in MSCs derived from WT and *Prrx1^Cre^ Mettl5^fl/fl^* mice. Compared with WT controls, *Mettl5*-deficient MSCs exhibited a significant reduction in anti-m^6^A antibody–mediated enrichment of 18S rRNA (Figure 3A). Similarly, silencing of *Mettl5* in MC3T3-E1 cells markedly decreased 18S rRNA m^6^A enrichment (Figure 3B). These findings collectively support that METTL5 is essential for the m^6^A modification of 18S rRNA. METTL5 is known to selectively regulate translation, and its KO leads to an overall reduction in translational activity, as previously described (38). To validate this effect in MSCs, we performed a puromycin incorporation assay, which showed a significant reduction in translation activity in *Mettl5-*KO MSCs compared with WT controls (Supplemental Figure 4). Furthermore, to investigate transcript-specific changes in translation efficiency upon *Mettl5* deficiency, we conducted ribosome profiling (Ribo-seq) in MSCs. A total of 232 mRNAs exhibited differential translation efficiency, including 97 transcripts with upregulated translation efficiency and 135 with downregulated translation efficiency in *Mettl5-*KO MSCs (Figure 3C and Supplemental Table 1). Notably, the translation efficiency of oxidative stress–responsive serine-rich protein 1 (*Oser1*) mRNA was reduced by approximately 16-fold in *Mettl5-*KO MSCs compared with WT controls.

To further examine whether METTL5 directly regulates *Oser1* translation, we constructed a dual-luciferase reporter plasmid containing the *Oser1* full-length sequence. In *Mettl5*-silenced MC3T3-E1 cells, the Firefly/Renilla luciferase ratio was significantly decreased, indicating impaired *Oser1* mRNA translation (Figure 3D). Consistent with this result, *Oser1* mRNA levels remained unchanged, whereas Western blot and immunofluorescence analyses revealed markedly reduced OSER1 protein levels in *Mettl5-*KO MSCs (Figure 3, E–G). Reduced OSER1 protein levels were also observed in MSCs derived from *Prrx1^Cre^ Mettl5^fl/fl^* mice and in MC3T3-E1 cells after *Mettl5* knockdown (Supplemental Figure 5).

### OSER1 contributes to osteogenic differentiation.

**Figure 4:**
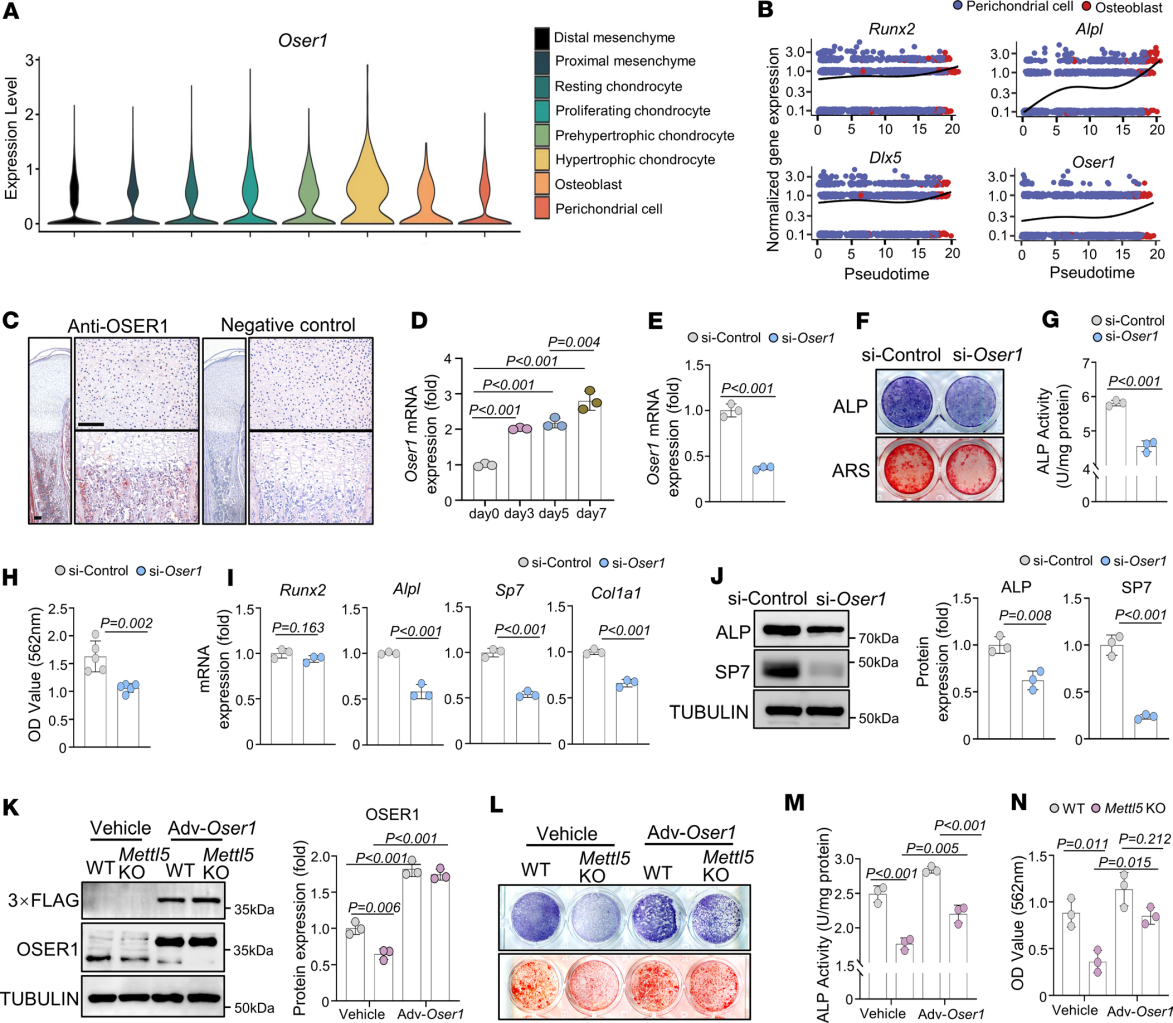
OSER1 is involved in osteogenic differentiation. (**A**) Violin plot showing *Oser1* expression levels in different cell types within skeletal cell populations of embryonic mouse limbs. (**B**) Pseudo-time analysis showing normalized expression of *Oser1* and osteogenic marker genes in perichondrial cells and osteoblasts along differentiation trajectory. (**C**) Representative images of IHC staining showing OSER1 expression in P1 mouse femurs. Scale bar: 100 μm. *n* = 3. (**D**) qRT-PCR analysis of *Oser1* mRNA levels at 0, 3, 5, and 7 days during osteogenic induction. *n* = 3. (**E**) qRT-PCR analysis of *Oser1* knockdown efficiency using siRNA in MC3T3-E1 cells. *n* = 3. (**F**) Representative images of ALP and ARS staining in si-Control and si-*Oser1*–treated MC3T3-E1 cells after osteogenic induction. *n* = 5. (**G** and **H**) Quantitative analyses of ALP activity (*n* = 3) and ARS staining (*n* = 5) in si-Control and si-*Oser1*–treated MC3T3-E1 cells after osteogenic induction. (**I**) qRT-PCR analysis showing expression of osteogenesis-related markers in MC3T3-E1 cells after osteogenic induction with si-Control or si-*Oser1* treatment. *n* = 3. (**J**) Representative Western blot images and quantifications showing protein levels of osteogenic markers in si-Control and si-*Oser1* MC3T3-E1 cells after osteogenic induction. *n* = 3. (**K**) Representative Western blot images and quantifications showing OSER1 protein levels in WT and *Mettl5-*KO MSCs transduced with OSER1-overexpressing or control adenovirus. *n* = 3. (**L**) Representative images of ALP and ARS staining in MSCs after osteogenic induction with OSER1-overexpressing adenovirus or control adenovirus. *n* = 3. (**M**) Quantitative analyses of ALP activity in MSCs after osteogenic induction with OSER1-overexpressing adenovirus or control adenovirus. *n* = 3. (**N**) Quantitative analyses of ARS staining in MSCs after osteogenic induction with OSER1-overexpressing adenovirus or control adenovirus. *n* = 3. Data shown as mean ± SD; *P* values determined by 1-way ANOVA (**D**), 2-tailed Student’s *t* test (**E** and **G**–**J**), and 2-way ANOVA (**K**, **M**, and **N**).

Given the marked reduction in *Oser1* translation efficiency upon *Mettl5* deficiency, we next investigated whether OSER1 contributes to osteogenic differentiation. OSER1 has recently been identified as a transcriptional target of FOXO, a key regulator of cellular defense against oxidative stress (39). However, its functional involvement in osteogenic differentiation has not been characterized. Analysis of publicly available single-cell RNA-seq data (ArrayExpress: E-MTAB-10514) from embryonic mouse limbs revealed that *Oser1* is broadly expressed across multiple skeletal cell populations, including mesenchymal, chondrocytic, and osteoblastic cells (40) (Figure 4A). Given the important role of perichondrial cells as a source of osteoblast progenitors during embryonic long bone development and their essential contribution to osteogenesis (41, 42), we examined the dynamic expression profile of *Oser1* during the differentiation of perichondrial cells into osteoblasts. *Oser1* expression increased in parallel with classic osteogenic markers such as *Runx2*, *Alpl*, and *Dlx5*, suggesting a potential involvement of OSER1 in osteogenesis (Figure 4B).

To further explore the role of OSER1 in osteogenesis, we examined its expression pattern in skeletal tissues. IHC staining of neonatal mouse femurs showed that OSER1 was detectable in resting zone chondrocytes as well as within the BM (Figure 4C). We then monitored its expression during osteogenic induction of MC3T3-E1 cells, which revealed an increase in *Oser1* mRNA levels during the differentiation process (Figure 4D). To assess the functional impact of *Oser1* deficiency, *Oser1* was silenced using siRNA in MC3T3-E1 cells (Figure 4E). Knockdown of *Oser1* significantly reduced ALP activity and mineralized nodule formation compared with controls (Figure 4, F–H). Consistently, qRT-PCR and Western blot analyses confirmed that *Oser1* knockdown decreased both mRNA and protein levels of key osteogenic markers (Figure 4, I and J). Notably, adenovirus-mediated overexpression of OSER1 in *Mettl5*-deficient MSCs partially rescued osteogenic differentiation, as evidenced by restored ALP activity and increased mineralized nodule formation (Figure 4, K–N).

### Mettl5 deletion decreases expression of key antioxidant genes.

**Figure 5:**
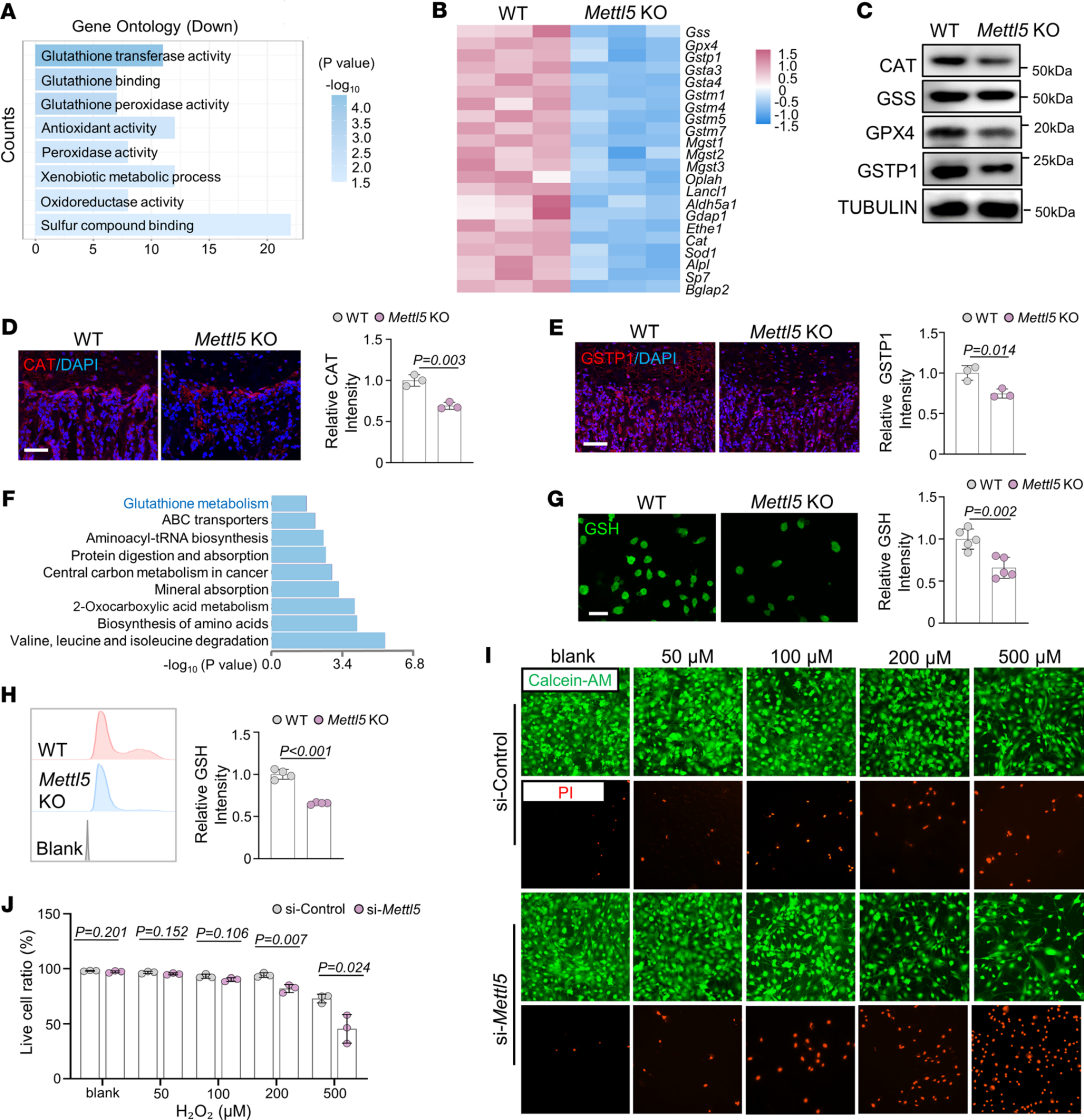
*Mettl5* deletion leads to reduced antioxidant capacity in osteogenic lineage cells. (**A**) GO enrichment analysis of downregulated genes in MSCs from *Mettl5-*KO mice after osteogenic induction. (**B**) Heatmap showing differentially expressed osteogenic and antioxidant-related genes identified by RNA-seq. (**C**) Representative Western blot images showing protein levels of selected antioxidant-related proteins in WT and *Mettl5-*KO MSCs. *n* = 3. (**D**) Representative immunofluorescence images showing CAT signal intensity and corresponding quantification in femurs from WT and *Mettl5-*KO mice. Scale bar: 100 μm. *n* = 3. (**E**) Representative immunofluorescence images showing GSTP1 signal intensity and corresponding quantification in femurs from WT and *Mettl5-*KO mice. Scale bar: 100 μm. *n* = 3. (**F**) KEGG pathway analysis of downregulated metabolites in the plasma of *Mettl5-*KO mice. (**G**) Representative fluorescent staining using a glutathione probe and corresponding quantification of glutathione signal in WT and *Mettl5-*KO MSCs. Scale bar: 40 μm. *n* = 5. (**H**) Flow cytometry analysis and quantification of glutathione levels in WT and *Mettl5-*KO MSCs. *n* = 4. (**I** and **J**) Representative images of Calcein-AM and PI staining in si-Control and si-*Mettl5* MC3T3-E1 cells treated with different concentrations of H_2_O_2_, with corresponding quantification of live cell ratios at each concentration. Scale bar: 100 μm. *n* = 3. Data are expressed as mean ± SD; *P* values were determined by 2-tailed Student’s *t* test.

To investigate the transcriptional changes in *Mettl5*-deficient MSCs, we performed RNA-seq on MSCs from *Mettl5-*KO and WT mice after osteogenic induction. RNA-seq identified 1,655 differentially expressed genes (DEGs), including 888 downregulated and 767 upregulated, in the *Mettl5-*KO group (adjusted *P* value < 0.05, |fold change| > 2). Gene Ontology (GO) analysis revealed substantial enrichment of downregulated genes associated with antioxidant activity, including those involved in glutathione transferase and glutathione peroxidase functions (Figure 5A). *Mettl5-*KO MSCs exhibited reduced expression of both osteogenic markers (*Alpl*, *Sp7*) as well as key antioxidant genes, including glutathione peroxidase 4 (*Gpx4*), glutathione S-transferase P1 (*Gstp1*), catalase (*Cat*), and superoxide dismutase 1 (*Sod1*), suggesting defects in both osteogenic differentiation and antioxidant defense (Figure 5B). These findings were validated by Western blot and immunofluorescence staining, which confirmed decreased levels of oxidative stress-related proteins both in vitro in *Mettl5*-KO MSCs (Figure 5C and Supplemental Figure 6A) and in vivo within the femurs of *Mettl5*-KO mice (Figure 5, D and E, and Supplemental Figure 6B). Despite the observed changes in translation efficiency, RNA-seq data showed comparable *Oser1* mRNA expression levels between the 2 groups (Supplemental Figure 6C).

To assess whether *Mettl5* deletion is associated with systemic alterations in antioxidant-related metabolism, we performed metabolomic profiling using plasma samples from *Mettl5-*KO mice and WT controls. Kyoto Encyclopedia of Genes and Genomes (KEGG) pathway analysis revealed a downregulated glutathione metabolism–related pathway in *Mettl5-*KO mice (Figure 5F). To assess intracellular antioxidant capacity in MSCs, we measured glutathione levels using fluorescence staining and flow cytometry. *Mettl5-*KO MSCs showed reduced intracellular glutathione compared with WT controls (Figure 5, G and H). To further evaluate the functional consequences of *Mettl5* deficiency on sensitivity to oxidative stress, we performed H_2_O_2_ challenge assays in MC3T3-E1 cells after siRNA-mediated knockdown of *Mettl5*. The si-Control and si-*Mettl5* cells displayed comparable viability under low concentrations of H_2_O_2_, but *Mettl5*-silenced cells exhibited markedly increased cell death at higher doses, indicating compromised oxidative stress resistance (Figure 5, I and J).

### Oser1 contributes to antioxidant defense in osteogenic lineage cells.

**Figure 6:**
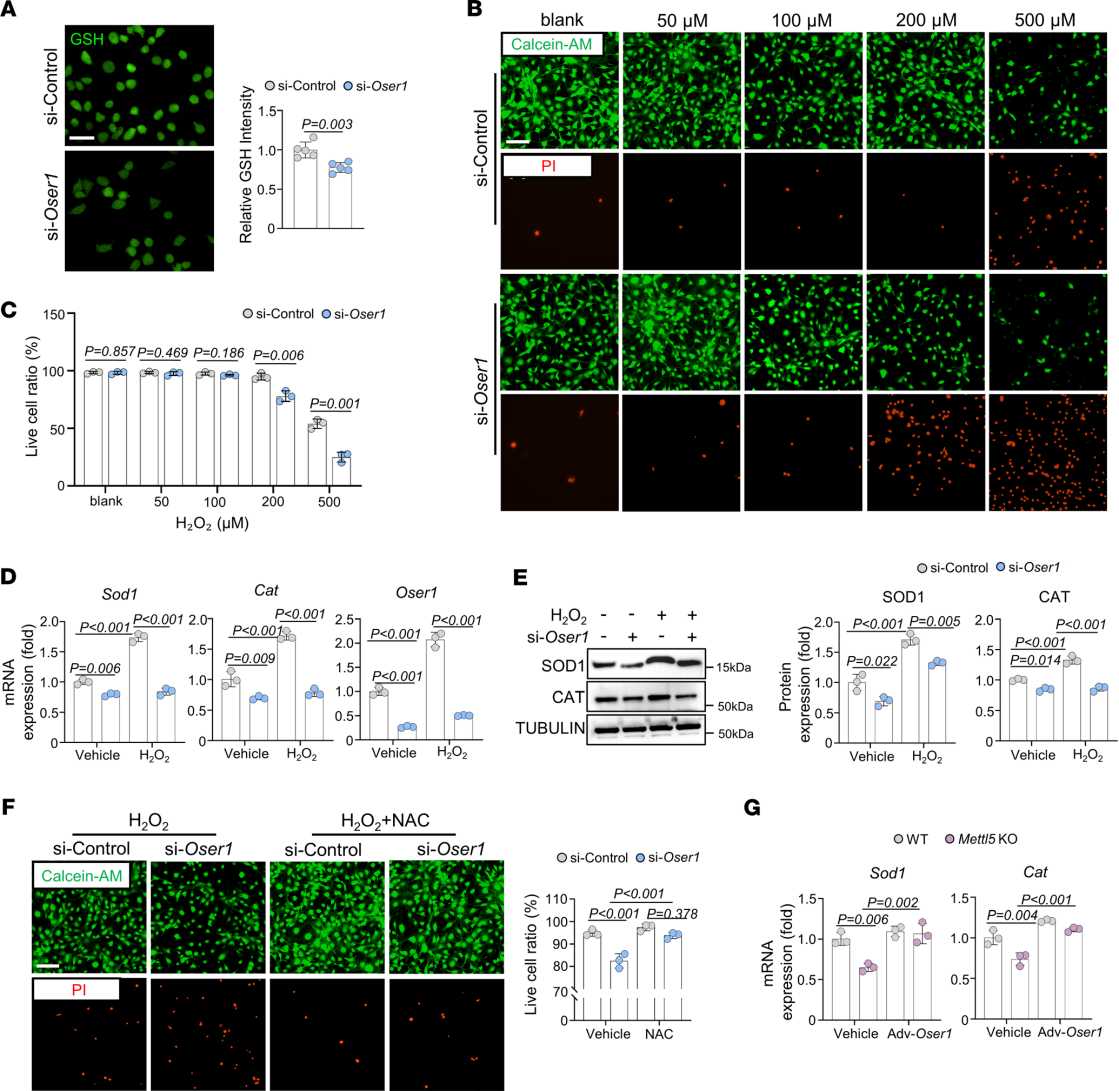
OSER1 supports antioxidant defense in osteogenic lineage cells. (**A**) Representative fluorescent probe staining and corresponding quantification of glutathione signal in si-Control and si-*Oser1* MC3T3-E1 cells. Scale bar: 40 μm. *n* = 5. (**B** and **C**) Representative images of Calcein-AM and PI staining in si-Control and si-*Oser1* MC3T3-E1 cells treated with increasing concentrations of H_2_O_2_, with corresponding quantification of the live cell ratios at each concentration. Scale bar: 100 μm. *n* = 3. (**D**) qRT-PCR analysis of antioxidant-related gene expression in si-Control and si-*Oser1* MC3T3-E1 cells treated with vehicle or 200 μM H_2_O_2_. *n* = 3. (**E**) Representative Western blot images and corresponding quantification showing antioxidant-related protein levels in si-Control and si-*Oser1* MC3T3-E1 cells treated with vehicle or 200 μM H_2_O_2_. *n* = 3. (**F**) Representative images and quantification showing the effects of N-acetylcysteine (NAC) treatment in si-Control and si-*Oser1* MC3T3-E1 cells after exposure to 200 μM H_2_O_2_, with or without NAC. Scale bar: 100 μm. *n* = 3. (**G**) qRT-PCR analysis of *Sod1* and *Cat* in WT and *Mettl5-*KO MSCs with vehicle treatment or adenovirus-mediated *Oser1* overexpression. *n* = 3. Data are expressed as mean ± SD; *P* values were determined by 2-tailed Student’s *t* test (**A** and **C**) and 2-way ANOVA (**D**–**G**).

To determine whether OSER1 contributes to antioxidant defense in osteogenic lineage cells, we examined the effects of *Oser1* loss on glutathione levels and sensitivity to oxidative stress. A previous study suggested that OSER1 is involved in the expression of antioxidant genes, such as *Cat* and *Sod1*, and that its deficiency can be partially rescued by antioxidant supplementation (39). Based on this information, we silenced *Oser1* in MC3T3-E1 cells and measured intracellular glutathione levels using a fluorescent probe. *Oser1* knockdown resulted in a marked reduction in glutathione content (Figure 6A). When exposed to increasing concentrations of H_2_O_2_, *Oser1*-silenced cells displayed comparable viability to control cells at low doses but exhibited significantly higher cell death at elevated H_2_O_2_ concentrations (Figure 6, B and C). Further analysis by qRT-PCR and Western blot demonstrated decreased expression of *Sod1* and *Cat* after *Oser1* knockdown. Notably, after exposure to 200 μM H_2_O_2_, control cells showed a significant induction of *Sod1* and *Cat* expression, whereas this adaptive antioxidant response was attenuated in *Oser1*-silenced cells, indicating compromised antioxidant response to oxidative stress (Figure 6, D and E). Supplementation with N-acetylcysteine (NAC), a glutathione precursor (43), reduced sensitivity to oxidative stress in *Oser1*-knockdown MC3T3-E1 cells (Figure 6F), consistent with a previous report that NAC partially reverses the *Oser1*-deficient phenotype (39). Furthermore, adenoviral overexpression of OSER1 in *Mettl5-*KO MSCs effectively restored the downregulated expression of *Sod1* and *Cat* (Figure 6G), indicating that OSER1 plays an important role in maintaining antioxidant defense in osteogenic lineage cells.

### NAC supplementation partially rescues the impaired osteogenesis in Mettl5-KO mice.

**Figure 7:**
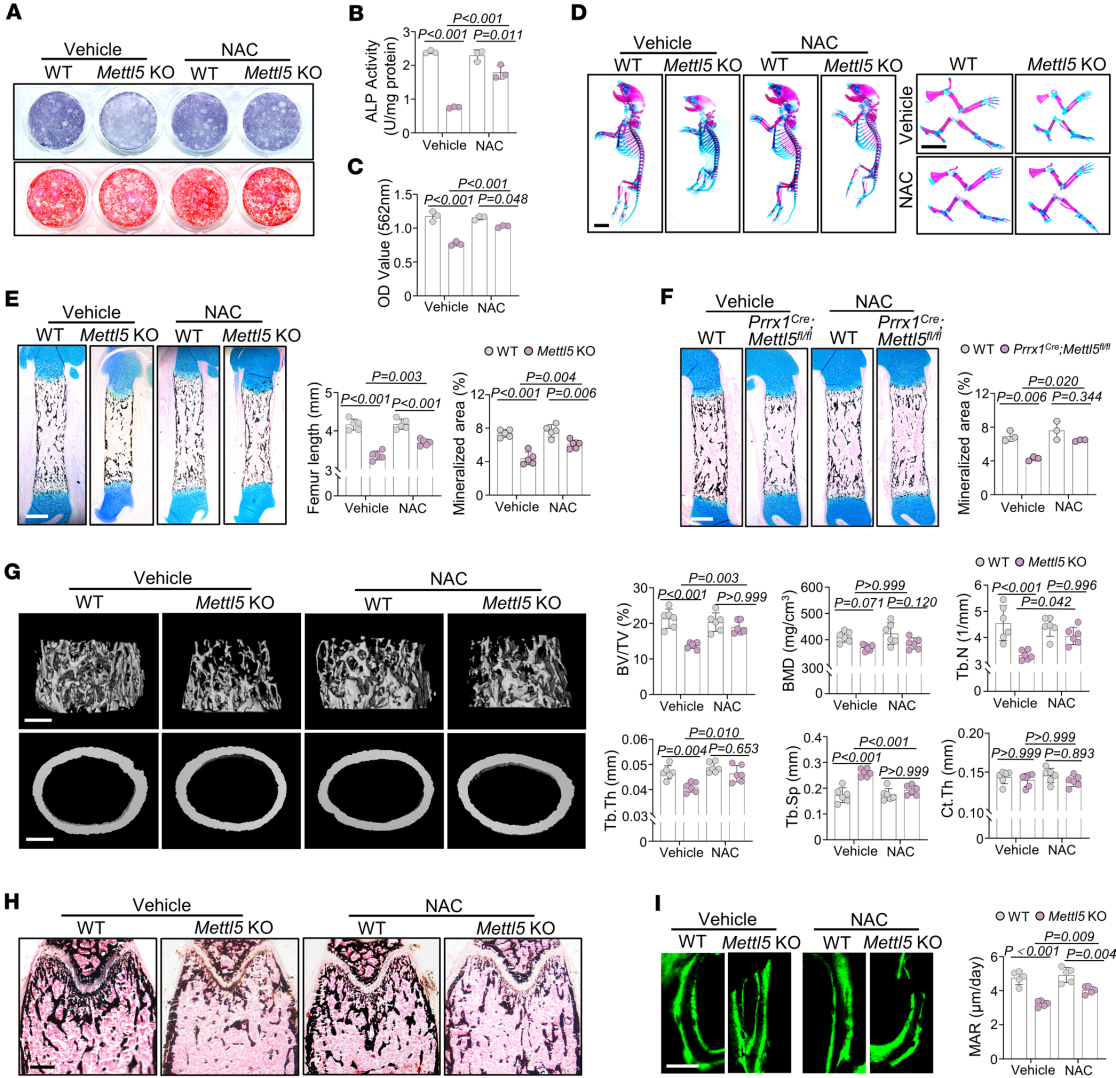
NAC supplementation partially rescues the impaired osteogenesis in *Mettl5-*KO mice. (**A**) Representative images of ALP and ARS staining in MSCs from WT and *Mettl5-*KO mice after osteogenic induction with vehicle or NAC treatment. *n* = 3. (**B** and **C**) Quantitative analyses of ALP activity and ARS staining in MSCs from WT and *Mettl5-*KO mice after osteogenic induction with vehicle or NAC treatment. *n* = 3. (**D**) Representative images of whole-mount skeletal staining in neonatal WT and *Mettl5-*KO mice after vehicle or NAC treatment. Scale bar: 4 mm. *n* = 3. (**E**) Representative Von Kossa staining and quantification of femur length and mineralized area of femurs from P1 WT and *Mettl5-*KO mice after vehicle or NAC treatment. Scale bar: 400 μm. *n* = 5. (**F**) Representative Von Kossa staining and quantification of mineralized area of femurs from P1 WT and *Prrx1^Cre^ Mettl5^fl/fl^* mice after vehicle or NAC treatment. Scale bar: 400 μm. *n* = 3. (**G**) MicroCT reconstructions of trabecular bone (top) and cortical bone (bottom) in femurs from 6-week-old male WT and *Mettl5-*KO mice after vehicle or NAC treatment, with corresponding quantitative analyses of trabecular and cortical bone parameters. Scale bar: 400 μm. *n* = 6. (**H**) Representative Von Kossa staining of femurs from 6-week-old male WT and *Mettl5-*KO mice after vehicle or NAC treatment. Scale bar: 400 μm. *n* = 3. (**I**) Representative Calcein double-labeling images of femoral trabecular bone from 6-week-old male WT and *Mettl5-*KO mice and corresponding quantification of mineral apposition rate (MAR). Scale bar: 50 μm. *n* = 5. Data are expressed as mean ± SD; *P* values were determined by 2-way ANOVA.

Given the reduced antioxidant capacity of *Mettl5-*KO MSCs, we investigated whether NAC supplementation could rescue the associated osteogenic defects. Treatment of *Mettl5*-deficient MSCs with NAC restored intracellular glutathione levels, as demonstrated by glutathione probe staining (Supplemental Figure 7). Furthermore, NAC treatment partially restored osteogenic differentiation capacity of *Mettl5-*KO MSCs, as evidenced by increased ALP activity and mineralized nodule formation (Figure 7, A–C).

To explore the effects of NAC supplementation in vivo, 2 administration protocols were used. For prenatal supplementation, NAC was provided in the drinking water of pregnant mice beginning at E10.5, and samples were collected at birth. For postnatal supplementation, NAC treatment was initiated after weaning and continued until 6 weeks of age, when samples were collected (Supplemental Figure 8A). Notably, embryonic NAC supplementation improved the growth phenotype of neonatal *Mettl5-*KO mice, resulting in body sizes comparable to those of control littermates (Supplemental Figure 8B). However, postnatal NAC administration initiated after weaning did not markedly rescue the body size difference between control and *Mettl5-*KO mice (Supplemental Figure 8C). Skeletal staining further demonstrated that NAC partially restored body and limb lengths in neonatal *Mettl5-*KO mice (Figure 7D). Von Kossa staining revealed increased femur length and mineralized area in NAC-treated *Mettl5-*KO mice (Figure 7E). Similarly, in neonatal *Prrx1^Cre^ Mettl5^fl/fl^* mice, bone mineralization was reduced compared with control littermates, and this defect was also partially rescued by NAC supplementation (Figure 7F). MicroCT analysis of femurs from 6-week-old male mice showed increased trabecular bone mass in NAC-treated *Mettl5-*KO mice (Figure 7G), which was supported by Von Kossa staining (Figure 7H). Moreover, dynamic bone formation analysis using Calcein double-labeling revealed enhanced mineral apposition rate in NAC-treated *Mettl5-*KO mice (Figure 7I).

## Discussion

In this study, we investigated the role of METTL5 in long bones and its impact on MSC differentiation. Our results demonstrate that *Mettl5* deficiency impairs osteogenic differentiation of BM MSCs by reducing antioxidant capacity, suggesting a crucial role for METTL5 in maintaining skeletal homeostasis through redox regulation.

Although clinical reports of individuals with METTL5 mutations primarily highlight developmental abnormalities such as short stature, craniofacial defects, and microcephaly, systematic evaluations of skeletal parameters have not been reported. Nevertheless, these phenotypes suggest a potential involvement of bone-forming processes in *Mettl5* deficiency. Our previous study using a *Mettl5-*KO model revealed delayed cranial suture closure accompanied by impaired osteogenic differentiation of cranial suture MSCs (12). Building on these observations, we further examined long bone phenotypes and found that *Mettl5-*KO mice exhibited reduced bone mass and overall growth, including shortened limb length. However, these growth defects were not observed in mice with limb mesenchyme–specific deletion of *Mettl5*. Since limb length is influenced by various systemic factors, it cannot serve as a direct indicator of osteogenic activity. Although no growth abnormalities were observed, *Prrx1^Cre^*-mediated deletion of *Mettl5* still resulted in marked defects in bone mass and bone formation. Consistent with these in vivo findings, *Mettl5*-deficient MSCs and *Mettl5*-silenced MC3T3-E1 cells both showed markedly impaired osteogenic differentiation in vitro. Collectively, these findings support a direct, cell-intrinsic role for METTL5 in osteogenesis, rather than an indirect effect of generalized developmental delay.

To explore the molecular basis underlying these phenotypes, we next examined the impact of *Mettl5* deficiency on translational regulation. Our findings indicate that *Mettl5* deficiency leads to an overall trend toward reduced translation efficiency, with *Oser1* showing selective translational downregulation. Notably, individual transcripts can respond to translational repression with unchanged, increased, or decreased translation efficiency. A previous study demonstrated that mRNAs containing G-quadruplex (G4) motifs or short 5′ UTRs are particularly susceptible to translational inhibition in the absence of *Mettl5* (44). Another report showed that transcripts harboring 5′ terminal oligopyrimidine (5′ TOP) motifs tend to display reduced translation efficiency in *Mettl5*-deficient cells (38). In the case of *Oser1*, although no canonical G4 or 5′ TOP motifs were identified, its relatively short 5′ UTR may account for its selective translational downregulation. Short 5′ UTRs can be more vulnerable to translational defects, particularly under conditions of reduced ribosome abundance (44). At present, this remains a working hypothesis. Further investigations, such as high-resolution analyses of RNA secondary structures and functional reporter assays incorporating 5′ UTR modifications, are needed to uncover the molecular mechanisms underlying this selective translational regulation.

Although METTL5 has been implicated in regulating cellular stress responses, the underlying mechanisms remain controversial. Loss of METTL5 has been associated with reduced translation of activating transcription factor 4 (Atf4), a key regulator of the integrated stress response, thereby impairing cellular adaptation to stress (36, 45). Conversely, work in *Caenorhabditis elegans* has indicated that METTL5 can enhance the translation of specific genes, such as *cyp-29A3*, leading to decreased stress resistance (37). In our study, *Mettl5* deficiency led to translational repression of *Oser1*, while antioxidant genes including *Sod1* and *Cat* were downregulated at the transcriptional level. Functionally, *Mettl5*-deficient MSCs displayed reduced resistance to oxidative stress. Supporting these findings, a recent study demonstrated that METTL5 modulates redox homeostasis by regulating glutathione metabolism through *Slc7a11* expression (46). Furthermore, *Mettl5* knockdown in hepatocellular carcinoma models led to transcriptional changes enriched in pathways related to FOXO signaling and glutathione metabolism, accompanied by decreased expression of antioxidant genes such as *Sod1* and *Cat* (47). These findings further align with the oxidative stress–related phenotypes observed in our study.

Antioxidants are crucial for maintaining bone homeostasis by regulating osteoblast and osteoclast differentiation. It is generally believed that activation of antioxidants promotes osteogenesis while inhibiting osteoclastogenesis (48, 49). Despite the dysregulation of the antioxidant system in *Mettl5*-deficient MSCs, the predominant effect was a decrease in osteogenic differentiation, with minimal effect on osteoclast differentiation. This finding challenges the conventional view that oxidative stress typically promotes osteoclast activity (50). A potential explanation lies in the complexity of antioxidant networks and their precise, dose-dependent regulation of osteoblast and osteoclast differentiation. Certain antioxidants, such as glutathione or NAC, have been reported to promote osteoclast differentiation under specific conditions (51–53), and compensatory mechanisms may stabilize osteoclastogenesis in the absence of METTL5. Therefore, the regulatory role of METTL5 in osteogenic and osteoclastogenic differentiation likely involves multiple regulatory mechanisms, and further studies are needed to clarify these complex interactions.

A recent study identified OSER1 as a novel regulator of lifespan and oxidative stress responses (39). Notably, knockdown of the OSER1 homolog in *Caenorhabditis elegans* was reported to induce mitochondrial fragmentation, a phenotype commonly associated with oxidative stress defects. Although mitochondrial dynamics were not examined in this study, this observation provides additional context for the antioxidant defects observed upon OSER1 loss. In our study, we show that *Oser1* deficiency impairs antioxidant capacity and stress responsiveness in MC3T3-E1 cells, as evidenced by reduced glutathione levels, impaired induction of antioxidant genes such as *Sod1* and *Cat*, and increased sensitivity to oxidative stress. These findings further support a role for OSER1 in maintaining cellular redox homeostasis in osteogenic lineage cells. Consistent with this notion, previous phenotypic data from the Mouse Genome Informatics (MGI) database indicate that *Oser1* mutant mice exhibit developmental abnormalities, including altered body size and increased neonatal mortality. Although our data support a functional link between OSER1 and antioxidant defense in osteogenic lineage cells, the precise mechanisms by which OSER1 influences redox regulation, as well as its potential contribution to skeletal development in vivo, remain to be determined. Further studies will be required to assess the bone phenotype of *Oser1* KO models and to elucidate whether OSER1 regulates antioxidant pathways directly or through intermediary factors.

Mutations in METTL5 have been linked to a recently identified syndrome characterized by moderate to severe intellectual disability, developmental delay, microcephaly, facial deformities, and behavioral abnormalities (8–10). Within the ClinVar database, multiple variants have been reported in the *METTL5* gene, including pathogenic and likely pathogenic variants and variants of uncertain significance, although skeletal involvement remains poorly understood. In this study, we administered NAC supplementation to *Mettl5-*KO mice and observed partial improvements in bone mass and growth-related parameters. Notably, these benefits were more pronounced when NAC was administered during embryonic development, highlighting the potential of antioxidant supplementation as a possible early intervention approach. It is also worth noting that NAC supplementation resulted in only a partial restoration of glutathione levels. This observation is consistent with the fact that NAC is converted intracellularly to cysteine and contributes to glutathione synthesis through the gamma-glutamyl cycle, rather than directly increasing intracellular glutathione (54). These observations provide a basis for further exploring antioxidant approaches in the context of METTL5-related disorders.

Moreover, plasma metabolomic profiling revealed that *Mettl5* deficiency disrupts multiple metabolic pathways, including branched-chain amino acid metabolism, 2-oxocarboxylic acid metabolism, amino acid biosynthesis, and glutathione metabolism. *Mettl5-*KO mice also exhibited smaller body size and lower body weight compared with control littermates (12, 55, 56). However, this phenotypic difference weakened with age, suggesting that METTL5 may have a stronger impact during early development, with partial compensation at later stages. Recent studies have further reported the metabolic regulatory mechanisms of METTL5. A marked reduction in fat mass has been observed in *Mettl5-*KO mice, accompanied by downregulation of genes involved in hepatic lipid synthesis and storage (56). This metabolic imbalance may result from the selective inhibition of mRNA translation related to lipid metabolism after METTL5 loss. Similarly, METTL5 has been shown to regulate fatty acid metabolism by modulating mRNA translation, thereby promoting hepatocarcinogenesis (38). Furthermore, recent studies have highlighted the role of METTL5 in controlling glucose and sphingomyelin metabolism (47, 57). Although these findings were primarily derived from cancer models, they collectively suggest that METTL5 maintains metabolic homeostasis at the translational level, which may also contribute to the developmental improvements observed with embryonic NAC supplementation.

In conclusion, the absence of METTL5 reduces the translation of *Oser1*, an important mediator of oxidative stress responses, thereby contributing to impaired antioxidant capacity and disrupted osteogenic differentiation. Importantly, antioxidant supplementation partially restores the osteogenic and developmental defects caused by *Mettl5* deficiency. Together, these findings support further evaluation of antioxidant-based interventions for *METTL5*-related disorders.

## Methods

### Sex as a biological variable.

In this study, both male and female mice were assessed, and comparable phenotypic characteristics were observed in both groups. However, to minimize variability due to sex differences, all data presented in this manuscript were derived from male mice.

### Mouse breeding.

The generation of *Mettl5^+/–^* mice and *Mettl5^fl/+^* mice has been previously described (12, 38), and Biocytogen Pharmaceuticals generated the mice used in this study. Homozygous *Mettl5^–/–^* (*Mettl5-*KO) mice were generated by intercrossing *Mettl5^+/–^* mice. *Prrx1^Cre^* and *LysM^Cre^* mice were purchased from The Jackson Laboratory (strains 005584 and 004781, respectively). All mice were maintained under specific pathogen–free conditions with a 12-hour light/12-hour dark cycle and provided with standard laboratory chow. The primer sequences used for genotyping are provided in Supplemental Table 2.

### Von Kossa staining.

Frozen sections were prepared from 6- or 12-week-old mice. Tissues were fixed in 4% paraformaldehyde (PFA) solution at 4°C for 24 hours on a shaker. After fixation, samples were immersed in 30% sucrose in PBS, embedded in OCT compound (4583, Sakura), and sectioned at 10 μm. Sections were incubated at 37°C for 1 hour, followed by sequential rinses in PBS and distilled water to remove OCT before staining.

Paraffin sections were prepared from neonatal mice. Tissues were fixed under the same conditions and, without decalcification, they were dehydrated, embedded in paraffin, and sectioned at 5 μm. Before staining, sections were deparaffinized and rehydrated through a graded ethanol series (100%, 90%, 80%, and 70%).

Von Kossa staining was performed on both frozen and paraffin sections using the same protocol. Sections were sequentially treated with 4% silver nitrate and 0.5% hydroquinone, followed by nuclear counterstaining with nuclear fast red. For neonatal mouse sections, Alcian blue solution (33864-99-2, Solarbio) was applied for 15 minutes to visualize cartilage.

### Immunofluorescence staining.

Samples were fixed in 4% PFA solution for 24 hours at 4°C on a shaker. After fixation, samples were decalcified using 10% EDTA (pH 7.4), and then dehydrated and embedded in paraffin. Paraffin sections (5 μm) were deparaffinized, rehydrated, and subjected to heat-induced antigen retrieval using a steamer in sodium citrate at 95°C for 15 minutes. The sections were cooled to room temperature and blocked with 5% BSA at 37°C for 1 hour. Primary antibody incubation was performed overnight at 4°C, followed by incubation with Alexa Fluor 647 goat anti-rabbit antibody (1:100, ab150079, Abcam) for 1 hour at room temperature. Nuclei were counterstained with 10 μg/mL DAPI (C0065, Solarbio) for 5 minutes. Images were taken using a fluorescence confocal microscope (FV3000, Olympus).

The primary antibodies used are listed as follows: rabbit anti-SP7 (1:250, ab209484; Abcam), rabbit anti-CAT (1:250, ER40125, Huabio), rabbit anti-GSTP1 (1:250, ET7107-71, Huabio), and rabbit anti-GPX4 (1:250, ET1706-45, Huabio).

### IHC staining.

Tissues were fixed in 4% PFA at 4°C for 24 hours on a shaker. After decalcification and dehydration, tissues were embedded in paraffin and sectioned at 5 μm. Sections were deparaffinized, rehydrated, and treated with 3% hydrogen peroxide to block endogenous peroxidase activity. Antigen retrieval was performed with sodium citrate buffer (pH 6.0) at 95°C for 20 minutes. After blocking with 5% BSA, sections were incubated overnight at 4°C with primary antibodies: METTL5 (1:250, 16791-1-AP, Proteintech) and OSER1 (1:250, CSB-PA865164LA01HU, Cusabio). The sections were then developed using the SABC kit (SA1028, Boster) and the AEC Substrate kit (AR1020, Boster). Counterstaining was performed with hematoxylin, and images were captured using a microscope (BX53, Olympus).

### MicroCT analysis.

A Venus MicroCT system (VNC-102) with a spatial resolution of 8 μm was applied to evaluate the bone parameters. The regions of interest (ROIs) were selected as previously described (58). Femurs from 6-week-old *Mettl5-*KO and *Prrx1^Cre^ Mettl5^fl/fl^* mice, along with their WT littermates, were analyzed, and femurs from 12-week-old *LysM^Cre^* *Mettl5^fl/fl^* mice and their WT littermates were used to perform analysis.

### Calcein double-labeling.

First, 6-week-old male mice were injected intraperitoneally with 20 mg/kg Calcein (154071-48-4, Sigma-Aldrich) on days 0 and 4. On day 6, the mice were euthanized, and the femurs were fixed, dehydrated, and embedded. The samples were sectioned at 10 μm, and the slides were imaged by a laser scanning confocal microscope (FV3000, Olympus). Quantitative analysis was performed with OsteoMeasure software (OsteoMetrics).

### Whole-mount skeletal staining.

The procedure for whole-mount skeletal staining was performed as previously described (58). Briefly, neonatal mice were eviscerated, the skin was removed, and the samples were fixed in 95% ethanol overnight. The samples were then treated with acetone for 24 hours and subsequently stained in Alcian blue solution (33864-99-2, Solarbio) overnight. After staining, the samples were washed using 70% ethanol and then placed in 95% ethanol overnight. After treatment with 1% potassium hydroxide solution for 1 hour at room temperature, the samples were stained with Alizarin red S (ARS) (130-22-3, Solarbio). The soft tissues were cleared using a series of solutions containing potassium hydroxide and glycerol at graded concentrations until clear skeletal profiles were obtained. Then, the samples were imaged using a stereomicroscope (SZX16, Olympus).

### Cell culture.

For extraction of BM MSCs, bilateral femurs and tibiae from 4-week-old mice were isolated. The BM cavity was thoroughly flushed using a 1 mL syringe with complete medium (α-MEM containing 10% FBS and 1% penicillin/streptomycin). The MSCs were cultured at 37°C in a humidified incubator with 5% CO_2_. After overnight culture, nonadherent cells were removed, and the adherent cells were refreshed with fresh complete medium. The mouse calvaria pre-osteoblast cell line MC3T3-E1 Subclone 14 (M7-0201, Cyagen Biosciences) was cultured under the same conditions.

For osteogenic induction, 50 μg/mL ascorbic acid (50-81-7, Sigma-Aldrich), 5 mM beta-glycerophosphate (154804-51-0, Sigma-Aldrich), and 100 nM dexamethasone (50-02-2, Sigma-Aldrich) were added to the complete medium. Cells were cultured in osteogenic medium for different durations depending on the downstream assays: 3 and 5 days for qRT-PCR; 5 days for RNA-seq; 7 days for ALP staining, ALP activity measurement, and Western blot; and 21 days for ARS staining and calcium nodule quantification. The medium was refreshed every 2–3 days.

To isolate BMDMs, nonadherent cells were collected the day after BM flushing. After erythrocytes lysis, the cells were cultured for 3 days in complete medium containing 50 ng/mL M-CSF (416-ML/CF, R&D Systems). For osteoclast differentiation, BMDMs were cultured in complete medium containing 50 ng/mL M-CSF and 50 ng/mL RANKL (462-TR/CF, R&D Systems) for 5 days to generate multinucleated osteoclasts.

### siRNA transfection.

siRNAs targeting *Mettl5* (sc-149389, Santa Cruz Biotechnology) and the corresponding negative control siRNA were purchased commercially. siRNAs specific for *Oser1* and negative control siRNA were designed and synthesized by RiboBio. When the cells reached 70%–80% confluence, transfections were performed using Lipofectamine RNAiMAX transfection reagent (13778150, Invitrogen) according to the manufacturer’s instructions. Transfection efficiency was evaluated 48 hours after transfection using qRT-PCR. The siRNA sequence targeting *Oser1* was 5′-CCAAGTCTCCCAAGAAAGT-3′.

### qRT-PCR.

Total RNA was extracted using TRIzol reagent (15596026, Invitrogen) and reverse-transcribed into cDNA using PrimeScript RT reagent kit with gDNA Eraser (RR047A, Takara) according to the manufacturer’s instructions. qRT-PCR was performed using SYBR Premix Ex Taq II (DRR041A, Takara) on a CFX96 Real-Time System (Bio-Rad). Relative gene expression was quantified using the 2^–ΔΔCt^ method, with *Gapdh* as the housekeeping gene for normalization. The primers used for qRT-PCR are listed in Supplemental Table 3.

### ALP staining and activity assays.

ALP staining on frozen sections was performed using an ALP staining kit (PMC-AK20-COS, Wako) according to the manufacturer’s instructions to visualize in vivo ALP activity.

For in vitro ALP assays, BM MSCs were cultured in osteogenic medium for 7 days. The cells were then fixed with 4% PFA for 15 minutes and stained using a BCIP/NBT staining kit (C3206, Beyotime). ALP activity was quantitatively measured with an ALP assay kit (P0321S, Beyotime) following the manufacturer’s instructions.

### ARS staining and quantification.

After 21 days of osteogenic induction, cells were fixed with 4% PFA for 15 minutes and stained with 1% ARS solution (130-22-3, Solarbio) for 20 minutes. After staining, 10% cetylpyridinium chloride solution was added to dissolve the calcium nodules, and calcium deposition was quantified by measuring the absorbance at 562 nm using a microplate reader.

### Western blot.

Western blot was performed as previously described (59, 60). Total cellular protein was extracted using a Total Protein Extraction kit (PE001, SAB), and protein concentration was determined using a BCA assay kit (P0010, Beyotime). Proteins were boiled with SDS Loading buffer (P0015, Beyotime) at 100°C for 5 minutes. Equal amounts of protein samples were separated on SDS-PAGE and transferred onto PVDF membranes (ISEQ00010, MilliporeSigma). After blocking with 5% skim milk (1172GR500, BioFroxx) for 1 hour at room temperature, the membranes were incubated with primary antibodies at 4°C overnight on a shaker. The following day, membranes were washed and incubated with HRP-conjugated secondary antibodies for 1 hour at room temperature. Finally, membranes were washed and visualized using a chemiluminescence detection system (E-Blot) according to the manufacturer’s instructions.

The primary and secondary antibodies used were as follows: rabbit anti-ALP (1:1,000, ET1601-21, Huabio), rabbit anti-SP7 (1:1,000, ab209484, Abcam), rabbit anti-osteopontin (1:1,000, HA723082, Huabio), rabbit anti-alpha-tubulin (1:2,000, AF5012, Beyotime), mouse anti-Puromycin (1:10,000, MABE343, MilliporeSigma), rabbit anti-CAT (1:1,000, ER40125, Huabio), rabbit anti-GSS (1:1,000, ET7107-62, Huabio), rabbit anti-GPX4 (1:1,000, ET1706-45, Huabio), rabbit anti-GSTP1 (1:1,000, ET7107-71, Huabio), rabbit anti-SOD1 (1:1,000, ET1702-36, Huabio), mouse HRP anti-DDDDK tag (1:5,000, ab49763, Abcam), rabbit anti-OSER1 (1:1,000, CSB-PA865164LA01HU, Cusabio), HRP-conjugated goat anti-rabbit IgG antibody (1:5,000, 7074, Cell Signaling Technology), HRP-conjugated horse anti-mouse IgG antibody (1:5,000, 7076, Cell Signaling Technology).

### Methylated RNA IP–qPCR.

m^6^A-modified RNA fragments were enriched using the EpiQuik CUT&RUN m^6^A RNA Enrichment kit (P-9018, Epigentek Group) following the manufacturer’s instructions. Briefly, total RNA was extracted from cells with TRIzol reagent (15596026, Invitrogen). For IP, 10 μg of total RNA was incubated with magnetic beads conjugated with either anti-m^6^A antibody or IgG control antibody. The enriched RNA was then fragmented, eluted, and purified with the provided magnetic beads, followed by reverse transcription into cDNA.

qPCR was performed with mouse 18S rRNA–specific primers. IgG IP was performed in parallel as a negative control to account for nonspecific background binding. Relative enrichment was quantified by the comparative ΔCt method, in which Ct values of each sample were normalized to those of the WT m^6^A group. ΔCt was defined as Ct (sample) – Ct (WT m^6^A), and relative enrichment was calculated as 2^−ΔCt^. Results are expressed as fold-enrichment relative to the WT m^6^A group.

### Puromycin incorporation assay.

MSCs were cultured to 80%–90% confluency and treated with 1 μM puromycin (HY-B1743, MedChemExpress) for 30 minutes in a 37°C incubator. After treatment, cells were harvested for protein extraction, and Western blot analysis was performed to assess puromycin incorporation in WT and *Mettl5-*KO MSCs.

### Ribo-seq.

Ribo-seq was carried out by Hangzhou Kaitai Biotechnology. To inhibit translation elongation, cycloheximide (100 μg/mL) was added to the culture medium, and cells were incubated for 2 minutes. The cells were lysed with lysis buffer containing 20 mM Tris·Cl (pH 7.4), 150 mM NaCl, 5 mM MgCl_2_, 1% (vol/vol) Triton X-100, 1 mM DTT, 25 U ml^−1^ Turbo DNase I, and 100 μg ml^−1^ cycloheximide (61). The lysate was then incubated on ice for 10 minutes and centrifuged to isolate the supernatant. To generate ribosome-protected fragments, RNase I was added to the lysate, followed by incubation at 10°C for 1 hour. The digested lysate was layered onto a sucrose solution and subjected to ultracentrifugation at 120,000*g* for 2 hours and 15 minutes using an MLA150 rotor. After discarding the supernatant, the pellet was resuspended in TRIzol reagent, and ribosome footprints were recovered through phase separation. RNA was purified, resuspended in nuclease-free water, and used for library preparation with the NEBNext Small RNA Library Prep Set for Illumina (E7300S, NEB). The libraries were then amplified, size-selected, and sequenced on the Illumina NovaSeq 6000 platform.

Translation efficiency was calculated by integrating Ribo-seq and RNA-seq. Both ribosome-protected fragments and RNA-seq reads were mapped to the coding sequence regions of protein-coding genes, and gene abundances were quantified using RSEM software with fragments per kilobase of transcript per million mapped reads (FPKM). Translation efficiency for each gene was defined as the ratio of Ribo-seq–derived FPKM to RNA-seq–derived FPKM. The transcripts with changed translation efficiency are listed in Supplemental Table 1.

### Luciferase reporter assay.

A dual-luciferase reporter plasmid was constructed by GeneChem by cloning the full-length sequence of mouse *Oser1* downstream of the Firefly luciferase (Fluc) coding sequence, with Renilla luciferase (Rluc) included as an internal control. MC3T3-E1 cells were transfected with the reporter plasmids using Lipofectamine 3000 (L3000015, Invitrogen) according to the manufacturer’s instructions. Cells were subsequently treated with si-Control or si-*Mettl5*. Luciferase activities were quantified with the Dual-Luciferase Assay System (E1910, Promega), and translational level was expressed as the Fluc/Rluc activity ratio.

### RNA-seq.

Total RNA was extracted from the MSCs using TRIzol reagent (15596026, Invitrogen). mRNA was enriched using polyT and then subsequently fragmented. The first-strand cDNA was reverse-transcribed, and second-strand cDNA synthesis was performed according to the Illumina standard protocol. Sequencing adaptors were ligated to the cDNA, and the library fragments were enriched by PCR. The samples were subjected to Illumina PE150 sequencing. Reads were mapped to the mouse genome (mm10) by Hisat2 (v2.0.5). Read counts were calculated by featureCounts, and differential gene expression analysis was performed using the DESeq2 R package (v1.20.0).

### Metabolomic assay.

Blood samples from 6-week-old mice were collected in anticoagulant-containing tubes, gently mixed, and centrifuged at 1,000 × *g* for 20 minutes at 4°C. The plasma supernatant was stored at –80°C for further metabolomic assays. Metabolomic assays were performed by Applied Protein Technology. Metabolites were extracted by adding a pre-cooled methanol/acetonitrile/water solution (2:2:1, v/v) to the plasma, followed by centrifugation. The supernatant was collected and vacuum-dried. Samples were dissolved in 100 μL of acetonitrile solution (1:1 acetonitrile/water, v/v), vortexed, centrifuged at 14,000*g* for 15 minutes at 4°C, and then the supernatant was collected. Metabolites were separated by ultra-high performance liquid chromatography (1290 Infinity LC, Agilent) and analyzed on an AB 6500+ QTRAP mass spectrometer (AB SCIEX).

### Glutathione probe assay.

Intracellular glutathione levels were detected by ThiolTracker Violet (T10095, Invitrogen). For fluorescence imaging, cells were incubated with 20 μM ThiolTracker Violet diluted in D-PBS with C/M (14287-080, Invitrogen) for 30 minutes at 37°C. After staining, cells were washed and fixed using 4% PFA for 15 minutes. Images were taken by a fluorescence confocal microscope (FV3000, Olympus). For flow cytometry, cells were detached with a cell scraper, centrifuged at 300 × *g* for 3 minutes, and resuspended in D-PBS with C/M containing 10 μM probe. After 30 minutes of incubation at 37°C, the staining solution was removed, and cells were resuspended in D-PBS with C/M for analysis by a flow cytometer (NovoCyte Advanteon, Agilent Technologies) with excitation at 405 nm.

### Calcein-AM and propidium iodide staining.

MC3T3-E1 cells were treated with H_2_O_2_ at concentrations of 0, 50, 100, 200, and 500 μM for 6 hours. After treatment, cells were stained using the Calcein/PI Cell Viability/Cytotoxicity assay kit (C2015M, Beyotime) according to the manufacturer’s instructions. Calcein-AM was used to stain viable cells; propidium iodide was used to label nonviable cells. Fluorescence images were captured using a microscope (LX73, Olympus).

### NAC supplementation.

For in vitro osteogenic induction, NAC (616-91-1, Sigma-Aldrich) was added at 100 μM from the beginning of induction until cell harvesting. For *Oser1* knockdown rescue, MC3T3-E1 cells transfected with si-Control and si-*Oser1* were cotreated with 200 μM H_2_O_2_ and 500 μM NAC for 6 hours before analysis. For in vivo experiments, NAC was supplied via drinking water at a concentration of 10 g/L.

### Cell immunofluorescence.

Cell immunofluorescence was conducted as previously described (62). Cells were seeded on coverslips and cultured until they reached approximately 60% confluence. They were then fixed with 4% PFA for 20 minutes, permeabilized with 0.5% Triton X-100 for 30 minutes, and blocked with 5% BSA for 1 hour at room temperature. After blocking, samples were incubated with rabbit anti-OSER1 antibody (1:1,000, CSB-PA865164LA01HU, Cusabio) at 4°C overnight. On the following day, coverslips were incubated with Alexa Fluor 647 goat anti-rabbit antibody (1:100, ab150079, Abcam) for 1 hour at room temperature, followed by nuclei counterstaining with DAPI (C0065, Solarbio). Fluorescence images were captured using a fluorescence confocal microscope (FV3000, Olympus).

### Adenovirus-mediated overexpression.

The adenovirus encoding 3×FLAG-tagged mouse *Oser1* and GFP was purchased from GeneChem. Primary MSCs were infected using MOI of 30 for 12 hours. The efficiency of overexpression was detected by Western blot 48 hours after infection.

### Statistics.

Statistical analyses were performed using GraphPad Prism (version 10.1.0). Data are presented as mean ± SD. For datasets labeled as “Relative expression,” the mean value of the WT or control group was defined as 1, and all individual values from both WT (or control) and experimental groups were expressed relative to this baseline. The number of replicates for each experiment is provided in the corresponding figure legends. Statistical significance was assessed using 2-tailed Student’s *t* test, 1-way ANOVA, or 2-way ANOVA, as indicated in the corresponding figure legends. A *P* value of less than 0.05 was considered statistically significant.

### Study approval.

All animal experiments were performed in accordance with the institutional guidelines of the Subcommittee on Research and Animal Care of Sichuan University.

### Data availability.

The raw sequencing data of RNA-seq, Ribo-seq, and metabolomics were deposited in the Genome Sequence Archive database under accession numbers CRA022545 (https://ngdc.cncb.ac.cn/gsa/browse/CRA022545), CRA022569 (https://ngdc.cncb.ac.cn/gsa/browse/CRA022569), and OMIX008865 (https://ngdc.cncb.ac.cn/omix/release/OMIX008865). Values for each data point presented in the graphs can be found in the Supporting Data Values file. Additional details necessary to reanalyze the data in this study can be obtained from the corresponding author upon reasonable request.

## Author contributions

KL, SK, SL, and Q Yuan conceived and designed the research studies. Q Yin, QL, FX, and SK developed the methodology. KL performed most of the experiments. QW, ZZ, and SL were involved in bioinformatic analysis. RX, XZ, and LP carried out data visualization. Q Yuan supervised the study. KL drafted the original manuscript. SK, SL, and Q Yuan reviewed and edited the manuscript. All authors read and approved the final version of the manuscript.

## Conflict of interest

The authors have declared that no conflict of interest exists.

## Funding support

- National Natural Science Foundation of China (82125006 and U24A20710 to Q Yuan).
- West China Hospital of Stomatology, Sichuan University (RCDWJS2025-2 to Q Yuan).

## Supplementary Material

Supplemental data

Unedited blot and gel images

Supporting data values

## Version 1 05/08/2026

Electronic publication
