# Supplementary material for: METTL5 deficiency impairs osteogenesis through OSER1-dependent antioxidant regulation
Source: JCI Insight. 2026 May 8;11(9):e194068. doi: 10.1172/jci.insight.194068 (PMC13232014; doi:10.1172/jci.insight.194068)
Supplement: Supplemental data [file jciinsight-11-194068-s049.pdf]

Supplemental data

METTL5 deficiency impairs osteogenesis through OSER1-dependent antioxidant regulation

Kexin Lei, Qi Yin, Qiwen Li, Qian Wang, Zhong Zhang, Fei Xue, Ruoshi Xu, Xinyi Zhou, Lin Peng, Shoichiro Kokabu, Shuibin Lin, Quan Yuan

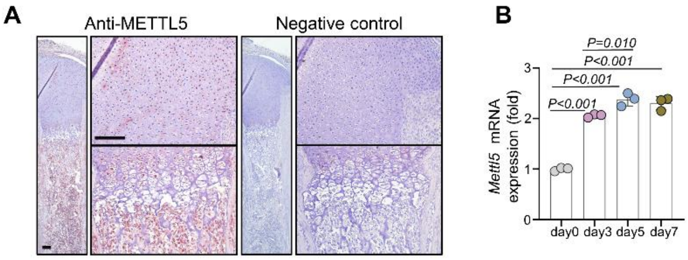

Supplemental Figure 1. METTL5 expression in bone tissues and during osteogenic differentiation of MC3T3-E1 cells.

(**A**) Representative images of immunohistochemical staining showing METTL5 expression in femurs from postnatal day 1 (P1) mice. Scale bar, 100 µm. n = 3.

(**B**) qRT-PCR analysis of *Mettl5* mRNA levels at day 0, 3, 5, and 7 during osteogenic induction of MC3T3-E1 cells. n = 3. Data are expressed as mean ± s.d.; P values were determined by one-way ANOVA.

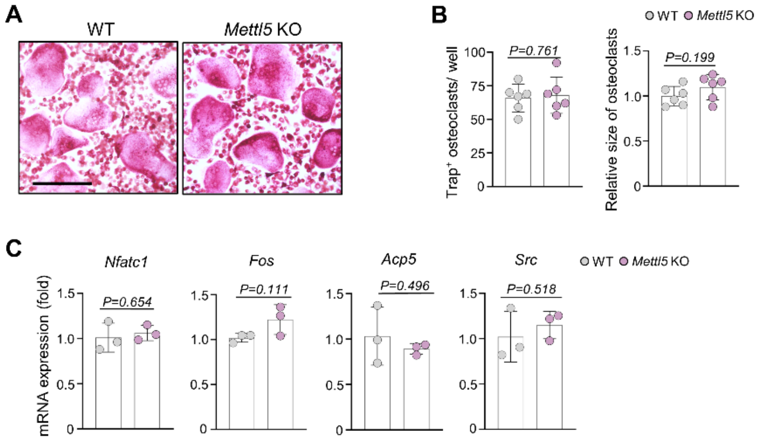

Supplemental Figure 2. ***Mettl5*** **deficiency does not affect osteoclast differentiation.**

(**A**) Representative TRAP staining images of osteoclasts differentiated from bone marrow–derived macrophages (BMDMs) isolated from WT and *Mettl5* KO mice. Scale bar, 200 µm. n = 6.

(**B**) Quantification of TRAP-positive osteoclast number (left) and relative osteoclast size (right) in WT and *Mettl5* KO groups. n = 6.

(**C**) qRT-PCR analysis of the expression of osteoclast markers in WT and *Mettl5* KO BMDMs after osteoclast differentiation induction. n = 3.

Data are expressed as mean ± s.d.; P values were determined by two-tailed Student's t test.

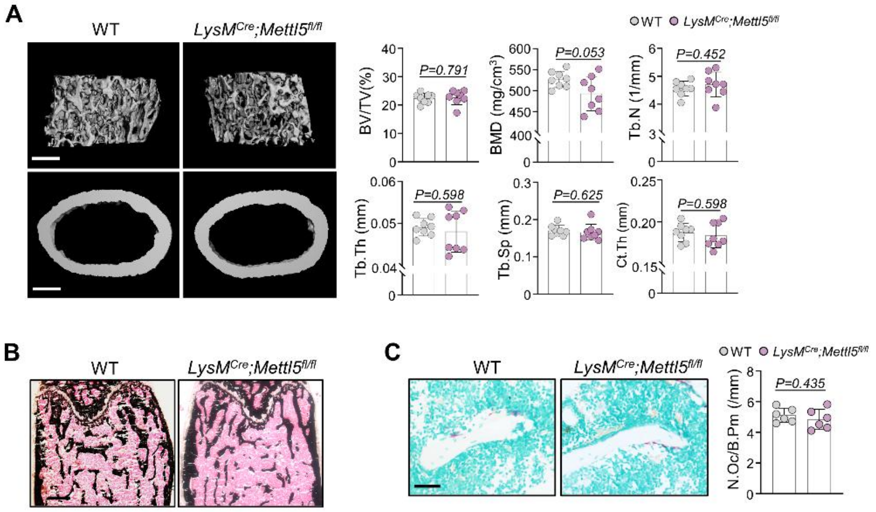

Supplemental Figure 3. Deletion of *Mettl5* in BMDMs does not affect skeletal phenotype.

(**A**) Representative microCT reconstructions of trabecular bone (top) and cortical bone (bottom) in femurs from 12-week-old male WT and *LysM*^{Cre}*;Mettl5*$^{fl/fl}$ mice, with corresponding quantitative analyses. Scale bar, 400 μm. n = 8.

(**B**) Representative Von Kossa staining of femurs from 12-week-old male WT and *LysM*^{Cre}*;Mettl5*$^{fl/fl}$ mice. Scale bar, 400 μm. n = 5.

(**C**) Representative TRAP staining images and corresponding quantification of osteoclast number per bone perimeter (N.Oc/B.Pm) in femurs from 12-week-old male WT and *LysM*^{Cre}*;Mettl5*$^{fl/fl}$ mice. Scale bar, 50 μm. n = 6.

Data are expressed as mean ± s.d.; P values were determined by two-tailed Student's t test.

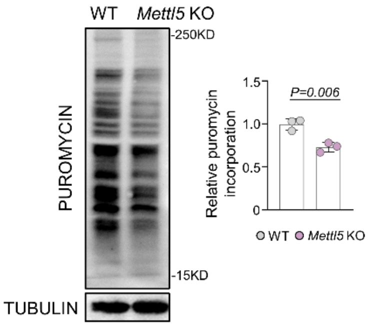

Supplemental Figure 4. *Mettl5* deficiency reduces global protein translation in MSCs.

Representative western blot images and quantification of puromycin incorporation in WT and *Mettl5* KO MSCs following puromycin treatment. n = 3. Data are expressed as mean ± s.d.; P values were determined by two-tailed Student's t test.

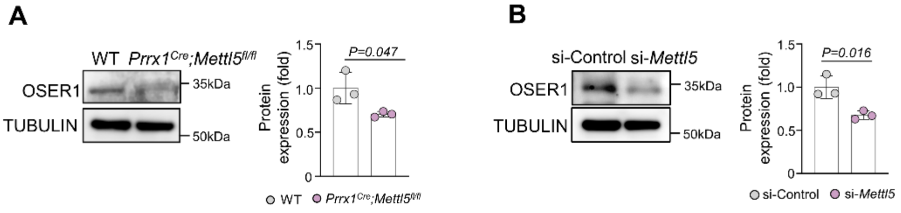

Supplemental Figure 5. Reduced OSER1 protein levels in *Mettl5*-deficient MSCs and MC3T3-E1 cells.

(**A**) Representative western blot images and quantification of OSER1 protein expression in MSCs from WT and *Prrx1*^{Cre}*;Mettl5*$^{fl/fl}$ mice. n = 3.

(**B**) Representative western blot images and quantification of OSER1 protein expression in MC3T3-E1 cells with si-Control and si-*Mettl5*. n = 3.

Data are expressed as mean ± s.d.; P values were determined by two-tailed Student's t test.

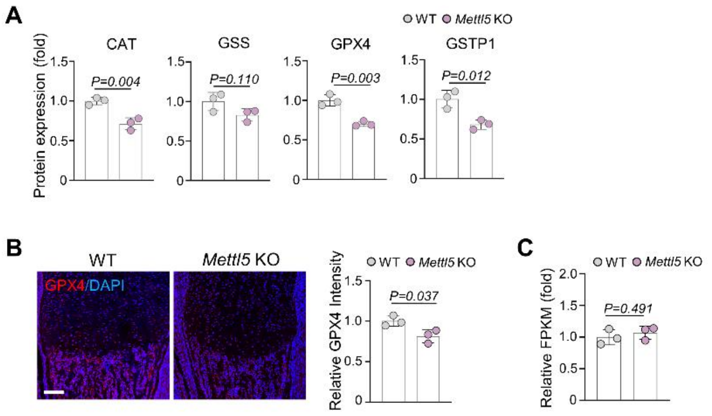

Supplemental Figure 6. Reduced antioxidant-related protein expression in *Mettl5*-deficiency.

(**A**) Quantification of western blot analysis showing the protein levels of CAT, GSS, GPX4, and GSTP1 in WT and *Mettl5* KO groups. n = 3.

(B) Representative immunofluorescence images and quantification of GPX4 expression in femurs from WT and *Mettl5* KO mice. Scale bar, 100 μm. n = 3.

(**C**) Fragments per kilobase of transcript per million mapped reads (FPKM) values of *Oser1* in WT and *Mettl5* KO MSCs from RNA-seq data. n = 3.

Data are expressed as mean ± s.d.; P values were determined by two-tailed Student's t test.

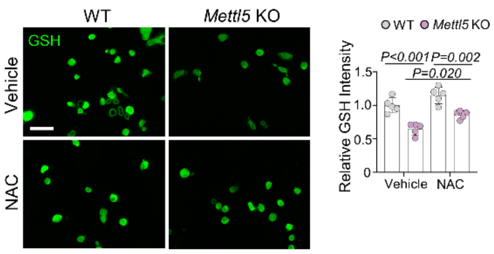

Supplemental Figure 7. NAC restores intracellular GSH levels in *Mettl5*-deficient MSCs.

Representative fluorescence images and corresponding quantification of intracellular GSH levels in MSCs from WT and *Mettl5* KO mice treated with vehicle or NAC. Scale bar, 40 µm. n = 5. Data are expressed as mean ± s.d.; P values were determined by two-way ANOVA.

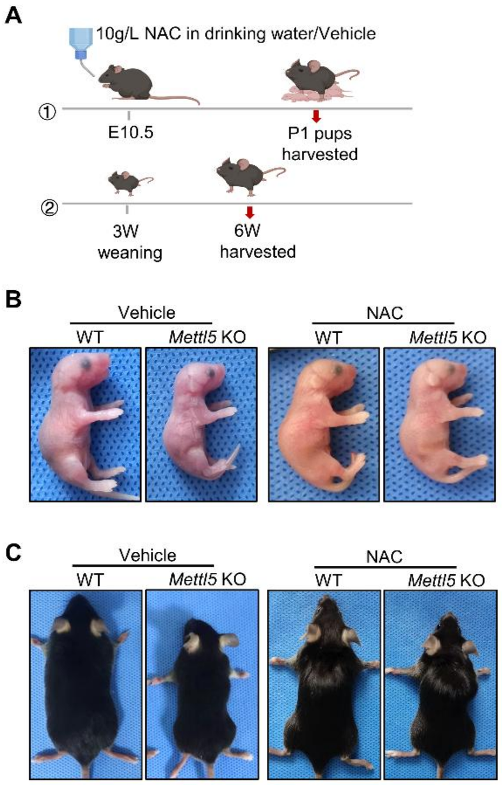

Supplemental Figure 8. Schematic of NAC administration and gross appearance of mice at sample collection.

(**A**) Schematic diagram illustrating NAC administration during the embryonic period and after weaning.

(**B**) Representative images showing the gross appearance of mice collected at the neonatal stage following NAC administration during the embryonic period. n = 8.

(**C**) Representative images showing the gross appearance of mice collected at 6 weeks of age following NAC administration after weaning. n = 6.

Supplemental Table 1. Differentially translated mRNAs identified by ribosome profiling in *Mettl5* KO and WT MSCs.

TE-down	log_2FoldChange	pvalue	TE-up	log_2FoldChange	pvalue
Galntl6	-6.02467	0.00151	*Rnf225*	1.009194	0.01213
Rasal2	-5.52585	0.006265	*Atg101*	1.012357	0.006775
Mdh2	-5.44291	0.000296	*Gadd45gip1*	1.022918	0.001988
Hoxb4	-4.9824	0.001441	*Ddx49*	1.053096	0.00013
Pdcl	-4.91271	0.001446	*Katnbl1*	1.072694	0.021405
Mettl5	-4.72657	0.002168	*Fos*	1.075616	0.01896
Klhl1	-4.59935	0.007948	*Ndufs2*	1.104818	0.029483
Uck2	-4.54495	0.003349	*Cep104*	1.113051	0.03273
Bcl9l	-4.42644	0.004382	*Dok2*	1.140878	0.03132
Bzw1	-4.33345	0.005124	*Tstd3*	1.164223	0.049492
Lrr1	-4.27633	0.00981	*Rexo1*	1.176781	0.021972
Cdc42bpa	-4.24483	0.007375	*Lyplal1*	1.189346	0.033951
Abhd16a	-4.23038	0.004798	*Mycbp*	1.231574	0.041217
Fzd9	-4.21749	0.011444	*Med20*	1.258916	0.009717
Plekhg6	-4.1918	0.015798	*Rras2*	1.273636	0.016933
Micu3	-4.17028	0.009495	*Pttg1ip*	1.276488	0.007649
Enho	-4.15402	0.010657	*Zc3h12a*	1.290659	0.03906
Tmeff1	-4.13444	0.008267	*P4ha1*	1.292545	0.001206
Pfkfb1	-4.12694	0.013656	*Fbxo36*	1.297356	0.019325
Med24	-4.12358	0.008902	*Dennd1a*	1.38151	0.045414
Borcs8	-4.04559	0.011373	*Cgrrf1*	1.479351	0.016152
Dio2	-4.02969	0.033468	*Fbln2*	1.581549	0.001046
Oser1	-4.02426	0.011724	*Cib2*	1.602212	0.041756
Mab21l3	-4.01055	0.022284	*Mboat1*	1.614163	0.010273
Ubash3b	-3.9696	0.014931	*Jazf1*	1.644151	0.03334
Gpc6	-3.9584	0.008574	*Tmem200b*	1.646147	0.024182

Thap2	-3.92293	0.014381	*Slc16a4*	1.652444	0.029069
Olfm1	-3.92175	0.01352	*Gas8*	1.657789	0.048918
Helb	-3.91713	0.014234	*L2hgdh*	1.682889	0.021385
Plce1	-3.91674	0.0163	*Mapk12*	1.704679	0.009332
Mapk6	-3.90804	0.015821	*Pms2*	1.734429	0.026502
Prdm11	-3.8389	0.017081	*Cptp*	1.812588	0.019894
Rnaseh1	-3.80916	0.017494	*Spin4*	1.827889	0.01435
Pank1	-3.80483	0.020981	*Adap1*	1.848776	0.016065
Pold1	-3.76646	0.01859	*Rab9*	1.931362	0.000189
Tent4a	-3.74326	0.021146	*Rab27b*	2.194518	0.022019
Dapk3	-3.7345	0.022246	*Cd84*	2.228248	0.0149
Bcl9	-3.70099	0.022576	*Arhgap1*	2.272895	0.04024
Ogfod2	-3.69752	0.021342	*Cdc25a*	2.342233	0.038872
Tnfaip2	-3.67851	0.023156	*C1qtnf1*	2.34767	0.01026
Sfswap	-3.67112	0.023311	*Alg10b*	2.5541	0.005765
Chuk	-3.66937	0.024203	*E2f2*	2.646111	0.027649
Jcad	-3.6633	0.016464	*Ostn*	2.714379	0.045899
Fndc3a	-3.65704	0.024002	*Bglap2*	2.899249	0.021291
Zc3h12d	-3.64787	0.02735	*Adora2b*	2.945809	0.019334
Tcerg1	-3.61754	0.016681	*Pias1*	2.95681	0.006721
Cadm4	-3.61056	0.040026	*Atp10d*	3.097882	0.041805
Ppia	-3.58333	0.017616	*Rgs14*	3.101724	0.006414
Usp19	-3.56997	0.027964	*Ulk1*	3.230128	0.046941
Senp8	-3.5332	0.031184	*Zc3h8*	3.233617	0.037784
Inhba	-3.52137	0.022601	*Ints10*	3.246062	0.046083
Spc25	-3.44777	0.034558	*Epc1*	3.265596	0.044466
Emx2	-3.40333	0.028425	*Csrp2*	3.31436	0.040368
Fbxw11	-3.33113	0.029889	*Sdc1*	3.322655	0.036269
Tmem140	-3.05625	0.049499	*Heatr3*	3.323783	0.040526

Plpp7	-2.83179	0.018808	*Pitpnm1*	3.34496	0.036393
St6galnac3	-2.71846	0.029262	*Ppp1r8*	3.349433	0.026417
Akr1c13	-2.6977	0.018102	*Rps15a*	3.352959	0.038375
Vit	-2.60761	0.005604	*Fam13c*	3.363362	0.038983
Tanc1	-2.60625	0.024198	*Cyb561d2*	3.393062	0.037362
Rab39	-2.51433	0.041171	*Prorsd1*	3.427937	0.022677
Dennd2a	-2.5031	0.033314	*Tgfb1i1*	3.429188	0.021301
Smarcb1	-2.45109	0.029623	*Sfn*	3.430903	0.0348
Gpsm3	-2.41179	0.037459	*Mmp8*	3.441674	0.047895
Dbr1	-2.38436	0.001879	*Cacul1*	3.448446	0.029918
Dnaaf5	-2.31073	0.01647	*Mypop*	3.49067	0.034068
Gigyf2	-2.2857	0.047726	*Mrps36*	3.507634	0.027784
Spns2	-2.26895	0.027985	*Zdhhc12*	3.531357	0.026677
Prss23	-2.20806	0.021702	*Atp6v0e2*	3.542613	0.039888
Wrap53	-2.20061	0.006528	*Polk*	3.571144	0.022408
Med22	-2.13356	0.004241	*Bod1l*	3.603999	0.021336
Rptor	-2.10453	0.030208	*Ereg*	3.632041	0.028508
Zfp760	-2.10152	0.005203	*Gap43*	3.639112	0.04465
Scmh1	-2.0452	0.034184	*Fam76a*	3.690142	0.018697
Pcdhb12	-2.02214	0.030047	*Rnf14*	3.715962	0.017594
2810021J22Rik	-1.97679	0.046886	*Rhd*	3.782378	0.042613
Lmo7	-1.96393	0.006297	*Mtif3*	3.786115	0.018594
Ube2g2	-1.96365	0.019227	*Pdcd7*	3.818063	0.016245
Klf7	-1.94953	0.021936	*Il1b*	3.838675	0.039791
Ropn1l	-1.9447	0.014043	*Gfod2*	3.850645	0.010676
Fbxl22	-1.94194	0.044541	*Gadd45b*	3.854027	0.012922
Arhgap10	-1.93534	0.047387	*Cracr2a*	3.863669	0.03053
Sema4f	-1.8737	0.037654	*Arpc5l*	3.87484	0.013576
Abcb9	-1.86767	0.027293	*Fgf21*	3.878917	0.017054

Smurf2	-1.80788	0.017039	*Tslp*	3.915572	0.014453
Lrfn3	-1.78223	0.003932	*Gamt*	3.976736	0.011299
AI429214	-1.77947	0.020949	*Gpr108*	4.001441	0.010024
Icam2	-1.77742	0.006128	*Lsm5*	4.056392	0.010154
Sirt2	-1.75684	0.038516	*Supt4a*	4.060049	0.009786
0610030E20Rik	-1.75156	0.003931	*Lsm6*	4.07055	0.009096
Prkacb	-1.73738	0.041366	*Rab33a*	4.155059	0.029172
Ctdspl	-1.69844	0.016477	*Wfs1*	4.186983	0.006188
Calr3	-1.6963	0.029705	*Cnot6l*	4.339389	0.004577
Fam72a	-1.69268	0.022088	*E2f6*	4.342703	0.004613
Plxna1	-1.69165	0.047959	*Tmem263*	4.392548	0.004136
Ubxn7	-1.68946	0.001542	*Dnah1*	4.676064	0.014787
Mtdh	-1.67436	0.029516	*Lrrn3*	5.942984	0.000395
Ric1	-1.66761	0.019583			
Rab5c	-1.66525	0.017457			
Zfp867	-1.65065	0.013476			
Syngr1	-1.61821	0.00355			
Utp14b	-1.60873	0.022428			
Ap2m1	-1.60784	0.005018			
Nkrf	-1.60613	0.001888			
Pcdhb5	-1.60577	0.002541			
Ubxn6	-1.58382	0.011057			
Kiz	-1.58203	0.00606			
Ncoa1	-1.56493	0.048073			
Akip1	-1.56481	0.017459			
Tmem9b	-1.55127	0.005225			
Cnn3	-1.52084	0.008614			
Lima1	-1.51111	0.000951			
Srsf3	-1.49355	0.025992			

D5Ertd579e	-1.43479	4.32E-05			
Zfp105	-1.4336	0.041938			
Fbxo45	-1.33789	0.009602			
Mnt	-1.27931	0.000752			
Sf3a2	-1.2442	0.025631			
Gbf1	-1.22121	0.015855			
Gm5617	-1.18447	0.020768			
Zfp526	-1.17863	0.024242			
Hipk3	-1.16654	0.011875			
Lpp	-1.14241	0.001775			
Slc22a5	-1.13302	0.046389			
Zc3h7a	-1.12089	0.006922			
Ctdnep1	-1.11297	0.000786			
Pcdhgb7	-1.10669	0.02457			
Aqr	-1.08242	0.043459			
Actn4	-1.06807	0.02294			
Itga2	-1.04456	0.004846			
Clspn	-1.03909	0.030685			
Atp2b4	-1.03827	0.0463			
Ccdc117	-1.03613	0.022289			
Gtpbp4	-1.00772	0.006652			
Irgq	-1.00574	0.016814			

Supplemental Table 2. Primers for genotyping.

Genotype	Primers	Sequence (5'-3')	Product size
Mettl5 WT	Forward	TGGGTTGTTAGGTTTGGCATTGAGC	WT = 552bp
	Reverse	CGCAATACCAGTGACTTCTCCTCCC	
Mettl5 Mut	Forward	TGGGTTGTTAGGTTTGGCATTGAGC	Mut = ~480bp
	Reverse	TGCTAATTCAGGACAGTCTGGGTCT	
Prrx1^{Cre} WT	Forward	ACTGGGATCTTCGAACTCTTTGGAC	WT = 397bp
	Reverse	GATGTTGGGGCACTGCTCATTCACC	
Prrx1^{Cre} Mut	Forward	CCATCTGCCACCAGCCAG	Mut = ~300bp
	Reverse	TCGCCATCTTCCAGCAGG	
LysM^{Cre}	Mut Reverse	CCCAGAAATGCCAGATTACG	WT = 350bp Mut = ~700bp
	Common	CTTGGGCTGCCAGAATTTCTC	
	WT Reverse	TTACAGTCGGCCAGGCTGAC	
Mettl5-flox	Forward	GCTTATATTCTGGCTTAGAATGACAGAC	WT = 239bp
	Reverse	ATAATTGGTATTTAGGAAGAGGCACC	Mut = 311bp

Supplemental Table 3. Primers for qRT-PCR.

Species	Gene	Forward	Reverse
Mus	*Gapdh*	AGGTCGGTGTGAACGGATTTG	TGTAGACCATGTAGTTGAGGTCA
Mus	*Alp*	AACCCAGACACAAGCATTCC	GCCTTTGAGGTTTTTGGTCA
Mus	*Bglap*	TTGGTGCACACCTAGCAGAC	ACCTTATTGCCCTCCTGCTT
Mus	*Sp7*	ATGGCGTCCTCTCTGCTTG	TGAAAGGTCAGCGTATGGCTT
Mus	*Runx2*	GGTACTTCGTCAGCATCCTATCAG	GCTTCCGTCAGCGTCAACAC
Mus	*Col1a1*	ACGCCATCAAGGTCTACTGC	TTCCGTACTCGAACGGGAATC
Mus	*Nfatc1*	GGAGAGTCCGAGAATCGAGAT	TTGCAGCTAGGAAGTACGTCT
Mus	*Fos*	CGGGTTTCAACGCCGACTA	TTGGCACTAGAGACGGACAGA
Mus	*Acp5*	CACTCCCACCCTGAGATTTGT	CATCGTCTGCACGGTTCTG
Mus	*Src*	GAACCCGAGAGGGACCTTC	GAGGCAGTAGGCACCTTTTGT
Mus	*Oser1*	AGCAAGCTCGTGTCTATGTGGAT	GGTGTACATCATCTCTGCCATGTG
Mus	*Sod1*	AACCAGTTGTGTTGTCAGGAC	CCACCATGTTTCTTAGAGTGAGG
Mus	*Cat*	AGCGACCAGATGAAGCAGTG	TCCGCTCTCTGTCAAAGTGTG
