# Supplementary material for: METTL5 deficiency impairs osteogenesis through OSER1-dependent antioxidant regulation
Source: JCI Insight. 2026 May 8;11(9):e194068. doi: 10.1172/jci.insight.194068 (PMC13232014; doi:10.1172/jci.insight.194068)

Full unedited gel for Figure 1N.

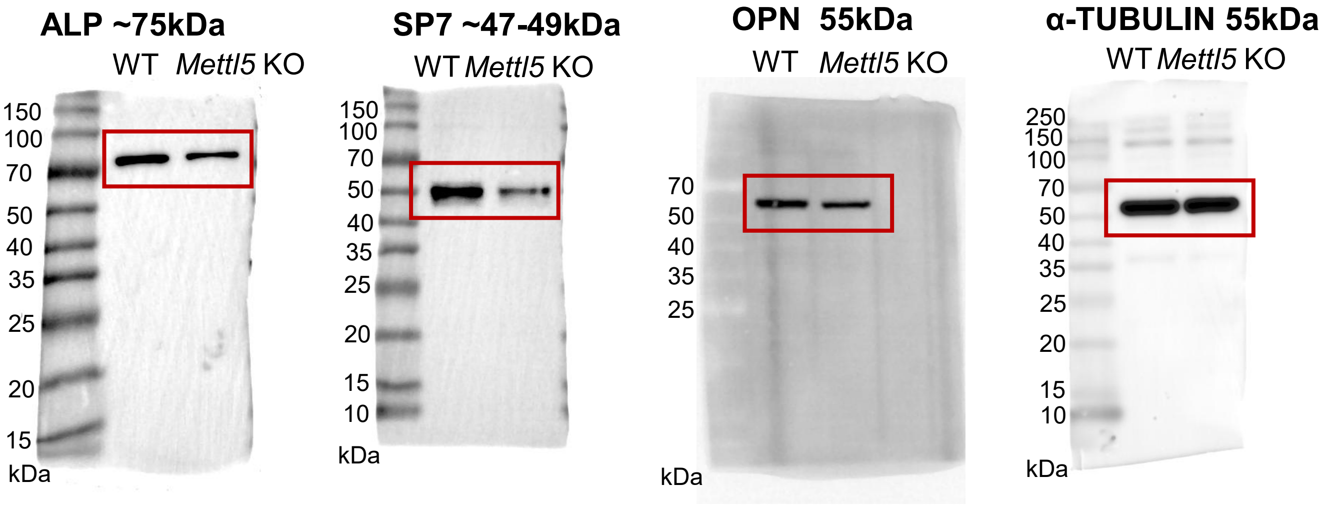

Full unedited gel for Figure 3F.

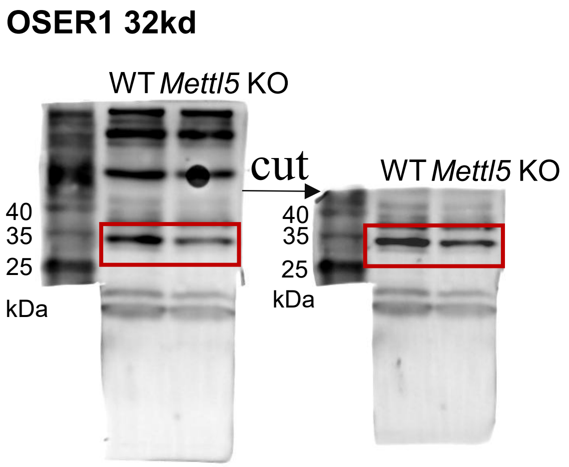

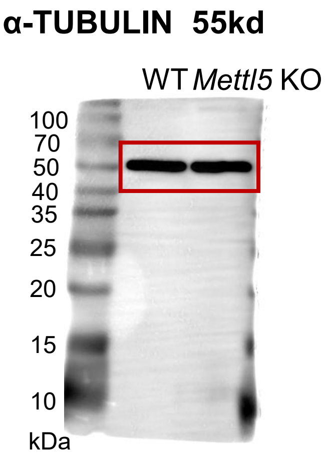

Full unedited gel for Figure 4J.

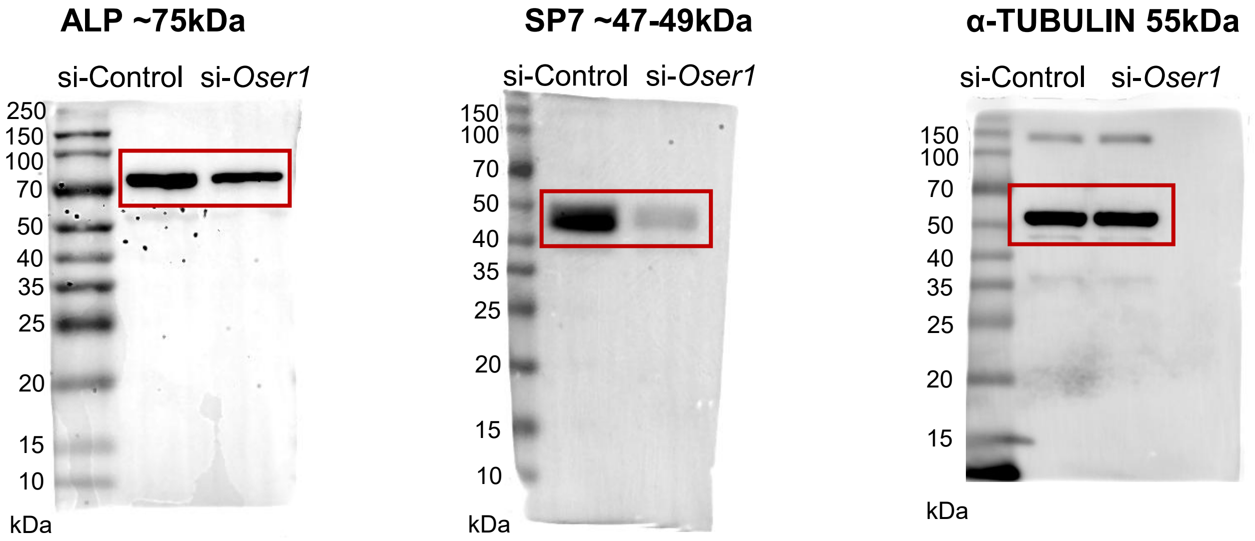

Full unedited gel for Figure 4K.

FLAG

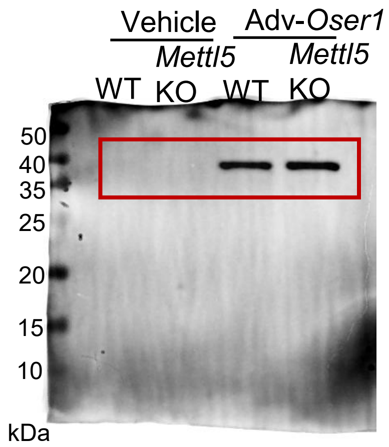

α-TUBULIN 55kd

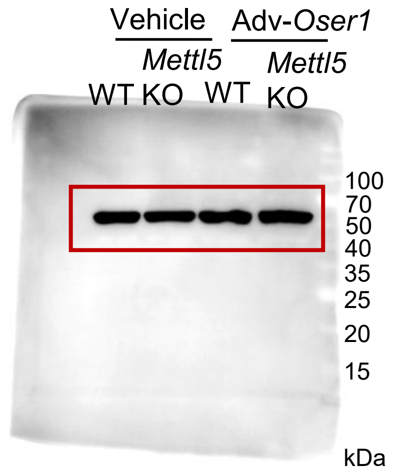

OSER1 32kd

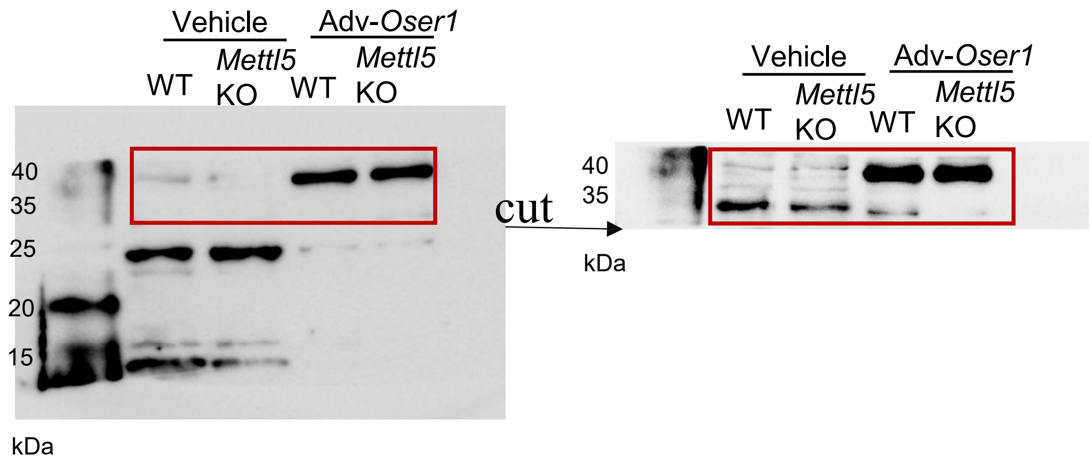

Full unedited gel for Figure 5C.

CAT 60kDa

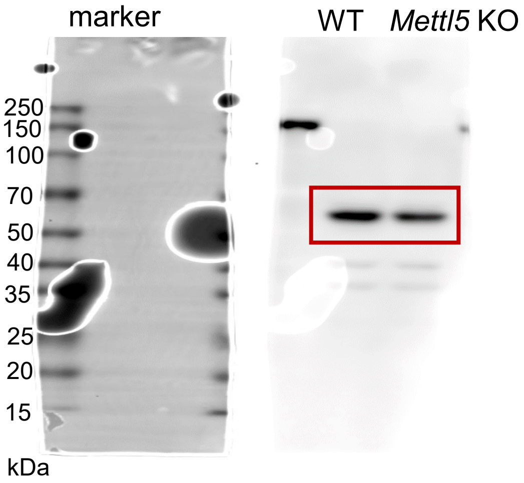

GSS 52kDa

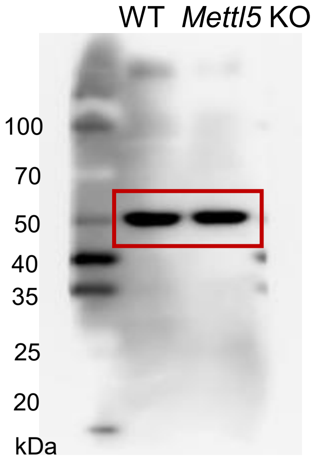

GPX4 17kDa

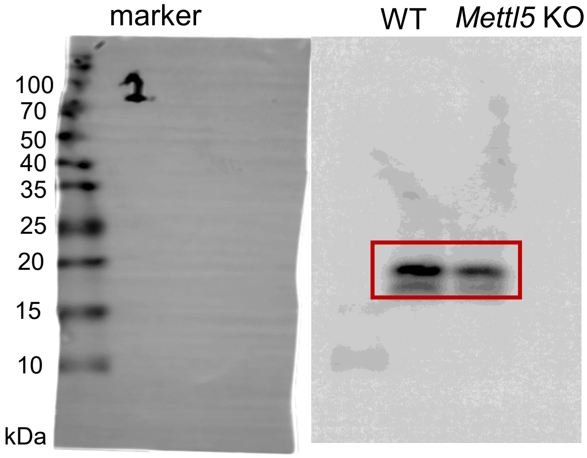

GSTP1 23kDa

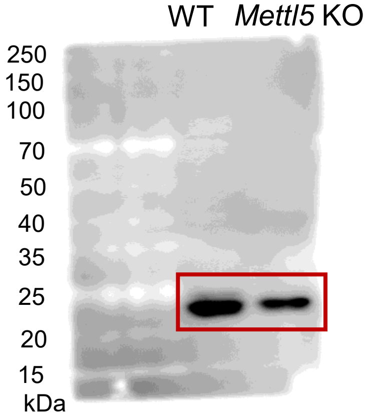

α-TUBULIN 55kDa

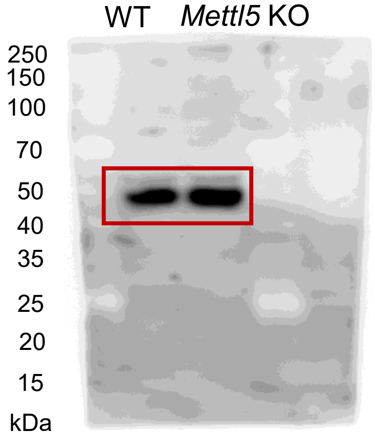

Full unedited gel for Figure 6E.

SOD1 ~16kDa

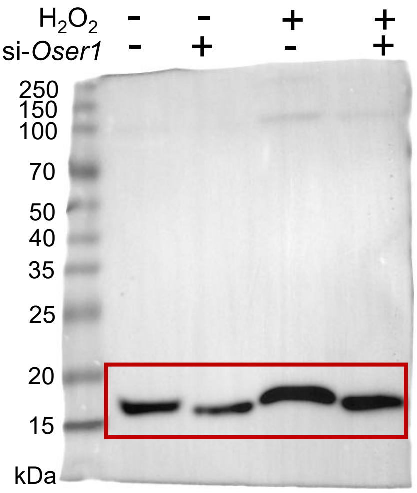

CAT 60kDa

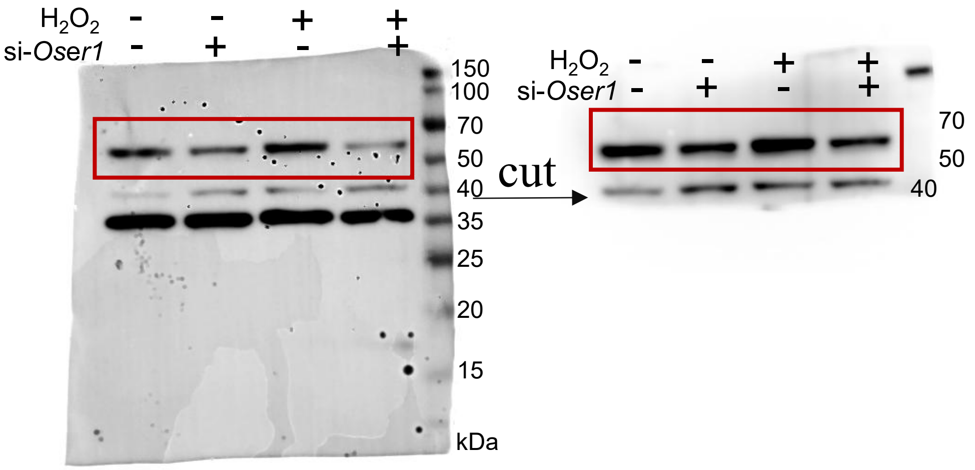

α-TUBULIN 55kDa

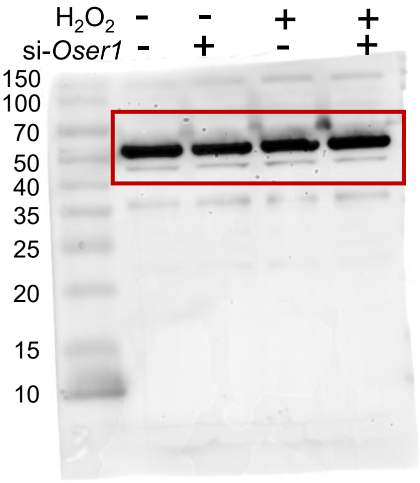

kDa

Full unedited gel for Supplemental Figure 4.

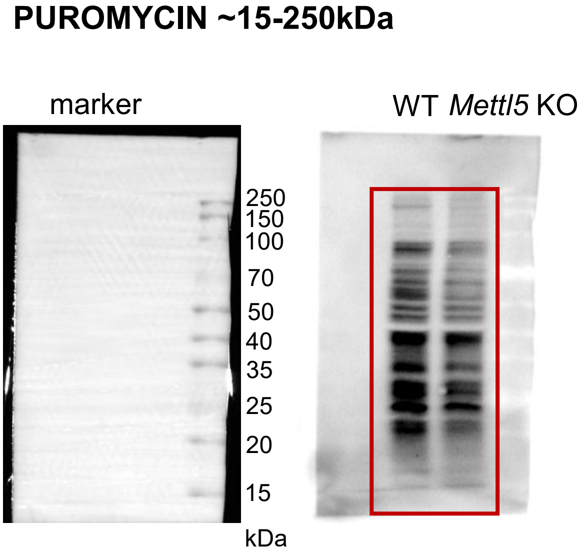

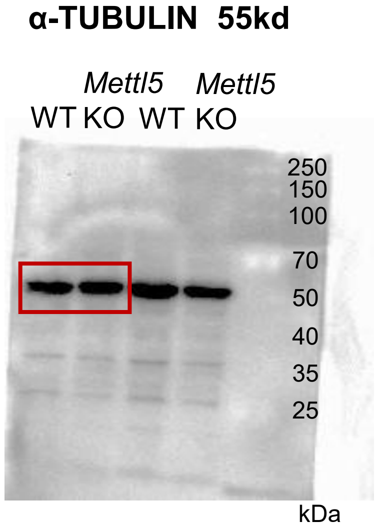

Full unedited gel for Supplemental Figure 5A.

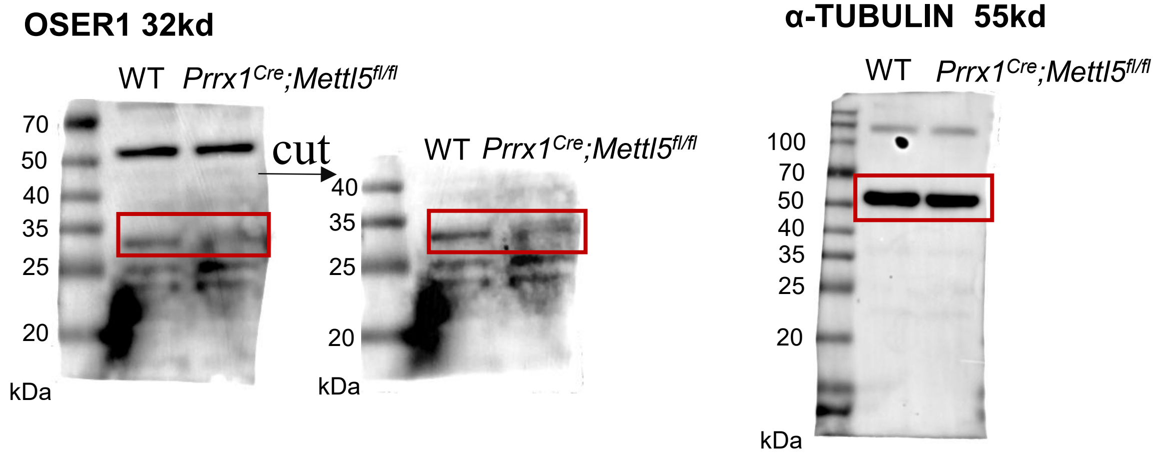

Full unedited gel for Supplemental Figure 5B.

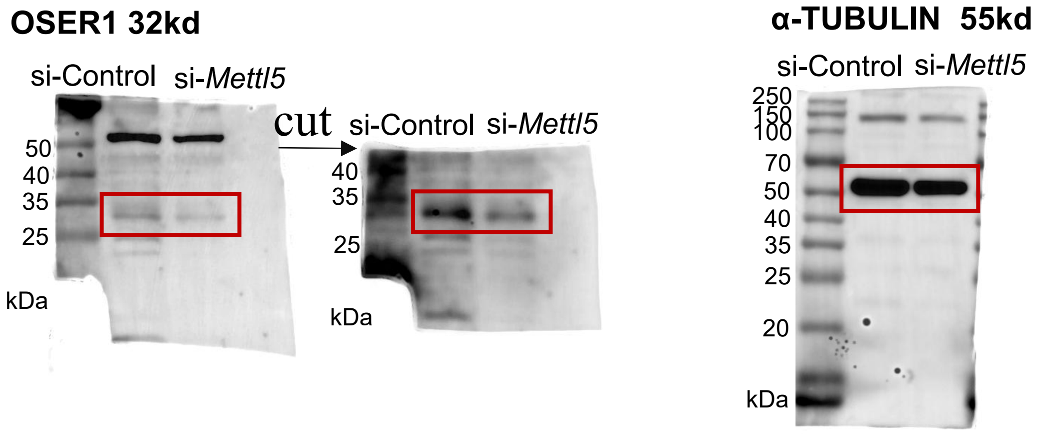

Supplement: Unedited blot and gel images [file jciinsight-11-194068-s050.pdf]
